# Exploring the biological basis for the identification of different syndromes in ischemic heart failure based on joint multi-omics analysis

**DOI:** 10.3389/fphar.2025.1641422

**Published:** 2025-07-28

**Authors:** Yilin Zhang, Jingjing Wei, Lijie Qiao, Rui Yu, Hongjie Ren, Anshe Zhao, Yang Sun, Aolong Wang, Bin Li, Xinlu Wang, Mingjun Zhu

**Affiliations:** ^1^ Department of Cardiovascular Disease, The First Affiliated Hospital of Henan University of Chinese Medicine, Zhengzhou, Henan, China; ^2^ The First Clinical Medicine College, Henan University of Chinese Medicine, Zhengzhou, Henan, China

**Keywords:** ischemic heart failure, multi-omics, biological basis, syndrome differentiation, traditional Chinese medicine

## Abstract

**Background:**

IHF is a major chronic disease that seriously threatens human health. Qi deficiency and blood stasis syndrome (QDBS), Yang deficiency with blood stasis syndrome (YDBS) and Yang deficiency and blood stasis with fluid retention syndrome (YDBSFR) are the basic syndromes of IHF in Chinese medicine. This study aims to explore the biological basis of the three IHF syndromes through integrated multi-omics research.

**Methods:**

We analyzed and integrated transcriptomic, proteomic, and targeted metabolomic data from IHF patients and healthy persons to obtain the key biomarkers and enriched pathways of QDBS, YDBS and YDBSFR(Registration No.: ChiCTR2200058314). These biomarkers were combined with clinical indicators to construct the “Disease-Syndromes-Clinical phenotypes-Biomarkers-Pathways” network, and the obtained differential genes and proteins were externally validated.

**Results:**

The potential biomarkers for QDBS included SDHD, IL10, ACTG1, VWF, MDH2, COX5A, Valeric acid, Succinic Acid and L-Histidine, which were predominantly enriched in TCA cycle, oxidative phosphorylation, platelet activation, and neutrophil extracellular trap formation pathways, demonstrating associations with energy metabolism, coagulation system, and immune-inflammatory responses.YDBS potential biomarkers included TSHR, PRKG1, ATP1A2, GNAI2, APOA2, PLTP, 3-Hydroxybutyrate, Hexadecanoic acid and Palmitelaidic acid, and the combined pathways were mainly enriched in thyroid hormone synthesis, regulation of lipolysis in adipocytes, cholesterol metabolism and PPAR signaling pathways, correlating with hormonal regulation and lipid metabolism. The potential biomarkers of YDBSFR were CNGB1, KCNMA1, PIK3R2, HSPA8, C3, FH, Oxamic acid, N-Acetyl-L-alanine, 4-Hydroxyhippuric acid, and the combined pathways were mainly enriched in aldosterone-regulated sodium reabsorption, cGMP-PKG signaling pathway, neutrophil extracellular trap formation and TCA cycle signaling pathways, which are related to hormone regulation, signal transduction, immune-inflammatory response and energy metabolism. Platelet activation was involved in the whole process of IHF. External validation demonstrated the above core targets.

**Conclusion:**

This study investigated the biological basis of QDBS, YDBS and YDBSFR in IHF from a modern biomedical perspective, providing references for the objective research of TCM syndrome differentiation.

## Graphical Abstract

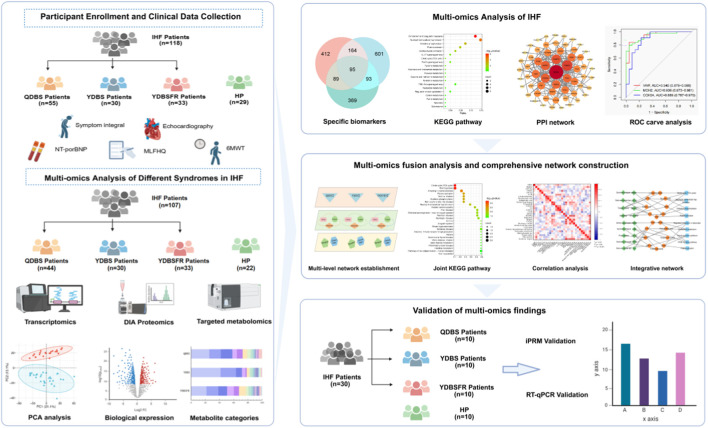

## Introduction

Heart failure (HF) is the terminal stage of various cardiovascular diseases and the most important cause of death. It has become a worldwide public health problem that seriously affects the health of residents. Its prevalence is constantly increasing due to population aging, complex comorbidities, and prolonged survival after myocardial infarction (Ponikowski et al., 2016).According to the Global Burden of Disease Study, there are currently about 64.34 million HF patients worldwide, with prevalence rates ranging from 1% to 3% in the general adult population (Savarese et al., 2023), and approximately 50% of HF patients are readmitted to the hospitals within 1 year after initial diagnosis (Lawson et al., 2019). Primary myocardial damage caused by ischemic heart disease (IHD), such as myocardial ischemia-hypoxia, infarction and scar formation, are the main causes of HF(Agarwal et al., 2024) and associated with readmission rates and high post-discharge mortality risk (Minicucci et al., 2011; Savarese et al., 2023). Traditional Chinese medicine (TCM) plays an important role in the treatment of IHF by intervening and treating the diseases according to the different stages of the diseases and the individual differences of the patients, i.e., “different treatments for the same disease”.

Syndrome differentiation and treatment is the characteristic and essence of TCM. Treatment selection is based on syndrome differentiation, so accurate syndrome identification is the prerequisite for TCM to maximize its therapeutic advantages. QDBS, YDBS and YDBSFR are the main TCM syndromes of IHF. Studies (Huang et al., 2023) have shown that QDBS is mostly seen in the early relatively stable stage of IHF, mainly of HFpEF type, with relatively low NT-proBNP levels, and its main clinical manifestations include easy fatigue after activity and spontaneous sweating; YDBS is mainly of HFrEF type, with higher NT-proBNP levels than QDBS, mostly New York Heart Association (NYHA) class III, and its main clinical manifestations include fear of cold and cold limbs; YDBSFR mostly occurs in the advanced stage, mainly NYHA class IV, with additional symptoms of dysuria and lower limb edema on the basis of the above clinical manifestations. Under the guidance of syndrome differentiation and treatment theory, TCM has played an important role in the prevention and treatment of cardiovascular diseases (Wang et al., 2023; Zhang et al., 2024). Exploring the biological basis of syndromes and elucidating the scientific nature of TCM syndrome differentiation in IHF is not only the key to objectifying TCM syndrome diagnosis, but also an important approach to improving the clinical efficacy of Chinese medicine and promoting TCM modernization.

Omics technologys can dynamically and efficiently characterize gene expression profiles in organisms and describe the interactions between multiple molecules, which is suitable for the study of complex TCM syndromes (Liu, 2009). In recent years, multi-omics correlation technologys guided by systems biology concepts have been increasingly applied in TCM research. For example, Wang et al. integrated metabolomics and proteomics analyses to identify seven differential metabolites, six differential proteins and six key pathways associated with dampness syndrome, establishing a symptom-centered diagnosis and treatment model for dampness syndrome (Wang et al., 2025). Wu et al. combined proteomics, metabolomics, and network pharmacology to reveal the biological differences between CCQS and QSBS of coronary heart disease in terms of potential biomarkers, biological processes, and comorbidities (Wu et al., 2021). These studies have played an important role in exploring the biological basis of TCM syndromes and improving the scientificity and objectivity of syndromic diagnosis. Current explorations of the biological basis of IHF symptoms are mostly conducted through single-omics studies such as genomics, proteomics or metabolomics (Liu et al., 2020; Luo et al., 2015; Meng and Wang, 2018). Due to the diversity of etiology and complexity of symptoms, single-omics studies ignore the crosstalk between molecules at different omics levels, which may lead to the omission of biological information to some extent (Liu et al., 2022). Using multi-omics fusion technology helps us understand molecular complexity at different levels. In this study, we included IHF patients with QDBS, YDBS, YDBSFR and HP, and conducted transcriptomics, data-independent acquisition (DIA) proteomics and targeted metabolomics on blood and urine samples to reveal differentially expressed genes, proteins and metabolites among the 3 syndrome types. After performing high centrality ranking combined with ROC curves to obtain potential biomarkers for QDBS, YDBS, and YDBSFR, respectively, and we further identified multi-omics joint pathways. Furthermore, we correlated the biomarkers with signs/symptoms and clinical indicators to construct the “Disease-Syndromes-Clinical phenotypes-Biomarkers-Pathways” network. Finally, we collected 10 QDBS, 10 YDBS, 10 YDBSFR patients of IHF and 10 HP for external validation to confirm key biological targets of the 3 syndromes.

## Materials and methods

### Participants recruitment

This study strictly followed the Declaration of Helsinki, and the protocol and informed consent forms were approved by the Ethics Committee of the First Affiliated Hospital of Henan University of Chinese Medicine (Approval No.: 2021HL-178). The informed consent was voluntarily signed by all participants, allowing public disclosure of their data while preserving their privacy rights. This study was registered at the Chinese Clinical Trial Registry in April 2022 (Registration No.: ChiCTR2200058314; https://www.chictr.org.cn/). It was conducted at the cardiology outpatient clinic of the First Affiliated Hospital of Henan University of Chinese Medicine from June 2022 to July 2023. Patients who meet all of the following criteria were eligible for enrollment: 1) aged 40–80 years; 2) IHF patients in accordance with the 2018 Chinese Heart Failure Diagnostic and Treatment Guidelines and the 2022 TCM diagnosis and treatment guidelines for chronic heart failure (The diagnostic criteria for syndrome types are shown in Supplementary Table S1); 3) left ventricular ejection fraction (LVEF) ≤ 50%,by modified Simpson method; 4) NYHA Class I–IV; 5) written informed consent obtained. Healthy adults with normal physical examinations will be included. Exclusion criteria include the presence of any of the following: 1) pulmonary embolism, acute coronary syndrome or acute cerebrovascular disease; 2) other cardiac diseases such as valvular heart disease, severe valve abnormalities, myocardial disease, congenital heart disease, or pulmonary heart disease; 3) liver and/or kidney dysfunction, malignant tumors or autoimmune diseases; 4) psychiatric disorders or substance abuse; 5) absolute contraindications to TCM; 6) pregnancy, planning for pregnancy or breastfeeding. The syndrome diagnosis of IHF patients and whether they met the inclusion and exclusion criteria were determined by Professor MJ Zhu, a renowned national expert, and verified by two professionally trained associate chief physicians (HJ Ren and AS Zhao).

### Clinical information collection

NT-ProBNP 3 mL of venous blood was drawn from the antecubital vein using heparin sodium anticoagulant tubes. After standing for 15 min, samples were centrifuged at 3,000 rpm for 3 min, and the supernatant was tested using a colloidal gold immunoassay kit (Getein Biotech, Nanjing, China).

Echocardiography A color Doppler ultrasound system (GE Vivid E95, United States) with an M5S transducer (frequency:1.7–3.4 MHz, frame rate: 40–80 Hz/s) was used. Two-dimensional Simpson’s method was applied to acquire 3–5 cardiac cycles from apical four-chamber and two-chamber views to measure LVEF, left ventricular end-diastolic dimension (LVEDD), left ventricular end-diastolic volume (LVEDV) and stroke volume (SV).

6-Minute Walk Test(6MWT) Conducted by researchers who have received standardized training. Subjects were asked to walk as fast as possible along a straight corridor to measure the 6-min walking distance (6MWD).

Quality of life assessment The Minnesota Living with Heart Failure Questionnaire (MLHFQ) was used to evaluate patients’ quality of life scores. The MLHFQ consists of 21 simple questions including physical, social, emotional and economic limitations, and each question is scored 0-5 (total 105 points). Higher scores indicate worse quality of life.

Laboratory indicators included blood lipids, glucose, and coagulation function parameters. TCM four diagnostic methods information was collected by cardiologists, recording symptoms/signs and syndrome integral for QDBS, YDBS, and YDBSFR patients.

### Transcriptomics study

Fasting morning venous blood of the subjects was collected in PAXgeneTM tubes (PreAnalytiX, China), thoroughly mixed, labeled, and gradient frozen according to the manufacturer’s instructions. Total RNA was extracted using PAXgene blood miRNA Kit (PreAnalytiX, China). RNA concentration and integrity were measured using Agilent 5400 Bioanalyzer (Agilent Technologies, United States). 150 bp paired-end sequencing was performed on Illumina NovaSeq 6000 high-throughput platform (Illumina, United States). FeatureCounts (v1.5.0-p3) was used to calculate gene expression levels. Supplementary Table S2 details the transcriptomic methodology. DESeq2 (V1.20.0) was used to normalize gene counts across samples, calculate fold change (FC), and adjust P-values using the Benjamini-Hochberg method to control false discovery rate (FDR). Differentially expressed genes (DEGs) were filtered at FC > 1.5 or FC < 0.67 with P < 0.05. Subsequently, Kyoto Encyclopedia of Genes and Genomes (KEGG) pathway enrichment analysis was performed using clusterProfiler (V4.12.0) based on the KEGG database (www.kegg.jp/kegg/pathway.html).

### Proteomics study

Fasting morning venous blood of the subjects was collected in EDTA anticoagulant tubes and gently inverted 8-10 times to fully anticoagulate. Samples were centrifuged at 3,000 *g* at 4°C for 15 min. The fresh supernatants were collected into Eppendorf tubes, labeled, and stored at −80°C. High-abundance proteins were removed using BioRAD ProteoMiner beads enrichment kit (Bio-Rad, United States). Protein concentration was measured using Bradford Protein Assay Kit (Beyotime, China).The samples were added to DB proteolytic solution, followed by trypsin digestion and TEAB buffer for enzymatic reaction and washed by C18 desalting column, and analyzed using liquid chromatography-tandem mass spectrometry (LC-MS/MS). Liquid mass detection was performed using an ultra-high performance liquid chromatography (UHPLC) system and the Orbitrap Astral mass spectrometer in DIA mode to acquire raw data for mass spectrometry detection. The raw data files were intensively searched and analyzed using the DIA-NN software. Supplementary Table S3 describes the DIA-based proteomics research method in detail. The criteria for selecting differentially expressed proteins (DEPs) were FC > 1.5 or FC < 0.67 with P < 0.05. Enrichment analysis of DEPs was performed using the Pathway database of KEGG.

### Targeted metabolomics study

Participants’ morning fasting venous blood of the subjects was drawn using EDTA anticoagulation tubes, thoroughly mixed and centrifuged. The supernatant was centrifuged at 3,000 *g* for 10 min at 4°C and then collected into Eppendorf tubes, labeled, and stored at −80°C. The samples were precipitated using aqueous methanol, derivatized, and then added to the internal standard solution to extract the metabolites for LC-MS detection. The metabolites were qualitatively and quantitatively analyzed using an ultra-high performance liquid chromatography-tandem mass spectrometry (UHPLC-MS/MS) system (ExionLC™AD UHPLC-QTRAP 6500+, AB SCIEX Corp., Boston, MA, United States). Supplementary Table S4 lists the detailed steps of targeted metabolomics. Data were processed and transformed using the metabolomics software metaX, and the variable importance projection (VIP) of metabolites was calculated by principal component analysis (PCA) and partial least squares discriminant analysis (PLS-DA). We used t-test to calculate the statistical differences of metabolites between the two groups, and also calculated the FC value. The criteria for identifying differential metabolites (DMs) were FC > 1.2 or FC < 0.833, VIP >1 and P < 0.05. The identified metabolites were annotated using KEGG database, HMDB database (https://hmdb.ca/metabolites) and LIPID MAPS database (http://www.lipidmaps.org/).

### Construction of protein-protein interaction (PPI) networks and screening of biomarkers

The DEGs and DEPs were input into the STRING database (https://string-db.org/) respectively with the minimum interaction score of 0.4 to construct PPI networks, which were visualized by Cytoscape (version 3.10.2). To identify genes/proteins with high centrality, we used the cytoHubba plugin to select the Maximum Clique Centrality (MCC), Neighborhood Component Centrality (MNC) and Degree algorithms to select the top 20 key genes/proteins and obtain the intersection of the three algorithms. ROC curves were then plotted to calculate AUC for comparing predictive accuracy, obtaining the top 3 candidate biomarkers for each syndrome.

### Joint analysis of transcriptomics, proteomics, and metabolomics

The key DEGs, DEPs, and DMs of QDBS, YDBS, and YDBSFR were input respectively into the “Joint-Pathway Analysis” module of MetaboAnalyst platform (https://www.metaboanalyst.ca/), with P ≤ 0.05 as the significance threshold, to identify enriched pathways of multi-omics molecules for each syndrome and clarify their co-involved key biological processes.

### Construction of the “Disease-Syndromes-Clinical Phenotypes-Biomarkers-Pathways” network

In the context of IHF, Spearman correlation analysis was performed to assess the relationships among symptoms, signs, NYHA classification, NT-proBNP, LVEF, LVEDD, LVEDV, SV, 6MWD, MLHFQ and core DEGs, DEPs and DMs of QDBS, YDBS, and YDBSFR patients. This revealed the associations between syndromes, clinical phenotypes, and biomarkers. Then, the results of multi-omics analysis of core biomarkers were combined with correlation analysis to obtain the intrinsic biological connections of the three syndromes from multiple levels and perspectives. We used Cytoscape visualization software to construct the “Disease-Syndromes-Clinical phenotypes-Biomarkers-Pathways” network to visualize the association between molecular markers and TCM syndromes.

### RT-qPCR and iPRM validation

We collected 10 QDBS, 10 YDBS, 10 YDBSFR patients of HIF and 10 HP to conduct independent external validation of the potential biomarkers. All the 40 subjects were from the cardiology outpatient clinic of the First Affiliated Hospital of Henan University of Chinese Medicine and met the inclusion and exclusion criteria of the main study. The real-time quantitative polymerase chain reaction (RT-qPCR) was conducted using BlazeTaq™ SYBR^®^ Green qPCR mix 2.0 (GeneCopoeia Green, United States), and the primer sequences listed in Supplementary Table S5. Samples were preprocessed, followed by phase separation, precipitation, washing, and RNA dissolution. RNA concentration and purity were measured by Nanodrop 2000. Reverse transcription reaction system was prepared and performed using a PCR machine. Then, PCR plates were used to prepare the reaction system, with 3 replicates per sample. After sealing and centrifugation, PCR quantification was performed using a fluorescence quantitative PCR instrument. Endogenous controls were normalized using endogenous regulatory genes (β-actin). Relative mRNA expression was calculated by the 2^−ΔΔCT^ method and repeated three times.

The intelligent parallel reaction monitoring (iPRM) technology was used to locate and quantify 9 target proteins in three syndrome types. Following strict SOP, samples were retrieved from −80°C for protein extraction and quantification. After passing quality control, samples were subjected to enzymatic digestion and desalting. Peptide information was acquired in DIA mode, generating raw mass spectrometry data files (.raw). Then, Spectronaut software was used for protein identification and quantitative analysis of the raw data. For each protein, 1-3 unique peptides were selected as candidate target peptides to ensure the accuracy and specificity of downstream analysis. When screening corresponding peptides of differential target proteins, peptides must be unique, without missed cleavage, without modifications other than alkylation, with charge state >1, and peptide length within 7–25, then export the list of the target peptide obtained from the screening. The chromatographic method is the same as the DIA data acquisition method. The obtained iPRM List was imported into the mass spectrometry acquisition method setup file to edit and form the iPRM acquisition method. The mass spectrometry acquisition mode was Full MS-PRM.

### Statistical analysis

Measurement data were described as Mean ± SD or Median (Q1, Q3), while count data were described by percentages or constituent ratios. Comparisons between multiple groups conforming to normal distribution were analyzed by one-way analysis of variance (ANOVA), and those not conforming to normal distribution were tested by the Kruskal-Wallis test. Spearman correlation analysis was used to clarify the relationships between key biomarkers and TCM syndromes and clinical indicators. P < 0.05 was considered as statistically significant.

## Results

### Baseline characteristics

From June 2022 to July 2023, this study recruited 118 IHF patients meeting inclusion and exclusion criteria at the First Affiliated Hospital of Henan University of Chinese Medicine, including 55 QDBS patients, 30 YDBS patients, and 33 YDBSFR patients, along with 29 healthy persons (HP) from the health examination center. The baseline demographic and biochemical characteristics of the four groups are shown in Table 1. Participants in the four groups were comparable in age, gender, BMI, heart rate, SBP, and DBP (P > 0.05). In the comparison of biochemical indicators, we found that the TC and LDL-C levels in the three syndrome types of IHF patients were lower than those in the HP group, indicating that the TC and LDL-C indexes in IHF patients were controlled within the normal range after standardized lipid-lowering therapy. Although the levels were higher in the HP group, they were still within the normal range, i.e., LDL <3.4 mmol/L and TC <5.2 mmol/L.

**TABLE 1:** Baseline demographic and biochemical characteristics of IHF and HP.

Characteristic	QDBS (N = 55)	YDBS (N = 30)	YDBSFR (N = 33)	HP (N = 29)	P value
Age, years	67.27 ± 7.68	66.4 ± 7.47	67.85 ± 8.52	64.97 ± 8.34	0.500
Male,N (%)	45 (81.82%)	23 (76.67%)	23 (69.70%)	19 (65.52%)	0.357
BMI,kg/cm^2^	24.26 ± 3.51	23.56 ± 2.84	23.34 ± 2.99	24.10 ± 3.57	0.559
HR,beat per minute	73.88 ± 3.55	70.33 ± 11.03	69.09 ± 11.12	68.86 ± 8.92	0.168
Seated SBP, mmHg	130.93 ± 22.86	129.77 ± 21.27	122.88 ± 15.60	119.90 ± 16.74	0.056
Seated DBP, mmHg	74.27 ± 14.16	72.5 ± 14.17	68.67 ± 9.98	75.34 ± 9.64	0.140
TC, mmol/L	3.73 ± 0.95	3.67 ± 1.03	3.69 ± 0.63	4.24 ± 0.53	0.023
TG, mmol/L	1.42 ± 0.56	1.34 ± 0.65	1.66 ± 1.13	1.24 ± 0.48	0.126
LDL-C, mmol/L	2.14 ± 0.68	2.11 ± 0.68	2.12 ± 0.54	2.63 ± 0.38	0.001
HDL-C, mmol/L	1.18 ± 0.25	1.18 ± 0.27	1.15 ± 0.25	1.26 ± 0.23	0.400
GLU, mmol/L	6.63 ± 2.32	6.55 ± 2.18	6.39 ± 2.19	5.15 ± 0.74	0.014
PT, s	12.42 ± 1.60	11.75 ± 2.13	12.47 ± 2.23	10.73 ± 0.68	<0.001
APTT, s	28.70 ± 3.40	28.68 ± 3.98	29.23 ± 4.25	30.92 ± 2.81	0.068
TT, s	15.21 ± 1.74	14.97 ± 1.55	15.18 ± 1.80	15.29 ± 0.96	0.885
FIB, g/L	3.42 ± 0.91	3.13 ± 0.91	3.27 ± 1.15	3.15 ± 0.56	0.445
Hypertension,n (%)	27 (49.09%)	13 (43.33%)	17 (51.52%)	0 (0.00%)	N/A
Diabetes mellitus,n (%)	16 (29.09)	8 (26.67%)	11 (33.33%)	0 (0.00%)	N/A
Hyperlipidemia,n (%)	7 (12.73)	4 (13.33%)	3 (9.09%)	0 (0.00%)	N/A

Data are Mean ± SD, or N (%).

Abbreviations: BMI, body mass index; HR, heart rate; SBP, systolic blood pressure; DBP, diastolic blood pressure; TC, total cholesterol; TG, triglycerides; HDL-C, high-density lipoprotein cholesterol; LDL-C, low-density lipoprotein cholesterol; GLU, glucose; PT, prothrombin time; APTT, activated partial thromboplastin time; TT, thrombin time; FIB, fibrinogen.

### Comparison of clinical indicators among the three syndromes

Intergroup comparison of the three clinical datasets revealed that (Table 2) with the evolution of QDBS, YDBS, and YDBSFR, NT-proBNP, LVEDD, LVEDV and MLHFQ scores showed progressively increasing trends. Except for LVEDV, all differences were statistically significant (P < 0.05). LVEF demonstrated a decreasing trend (P < 0.001), and 6WMD gradually decreased (P = 0.017). In terms of NYHA classification, QDBS was predominantly class II (70.91%), YDBS showed the highest proportion of class III (50.00%), and YDBSFR was also mainly class III (48.48%), with statistically significant differences (P < 0.001). Additionally, we observed that the proportion of class IV patients in YDBSFR (21.21%) was significantly higher than in QDBS (3.64%) and YDBS (6.67%). With the evolution of QDBS, YDBS and YDBSFR, the condition of IHF progressively worsens.

**TABLE 2:** The differences in cardiac function indicators among the QDBS, YDBS and YDBSFR.

Indicator		QDBS (N = 55)	YDBS (N = 30)	YDBSFR (N = 33)
NT-proBNP(pg/mL)		736 (510,1470)	1210 (798.75,1581.25)	1419 (1137,3757.5)
LVEF (%)		42.11 ± 5.50	40.13 ± 7.47	35.36 ± 8.28
LVEDD (mm)		53.78 ± 7.102	55.4 ± 5.703	59.24 ± 8.842
LVEDV (ml)		117.8 ± 51.135	120.48 ± 49.146	143.45 ± 62.15
SV(ml)		54.53 ± 20.792	46.76 ± 18.675	47.99 ± 19.06
6MWD(m)		377.98 ± 93.90	365.53 ± 77.11	311.91 ± 139.07
MLHFQ		43.42 ± 11.27	46.63 ± 15.28	56.39 ± 14.98
NYHA classification	I	2 (3.64%)	0 (0%)	0 (0%)
	II	39 (70.91%)	13 (43.33%)	10 (30.30%)
	III	12 (21.82%)	15 (50.00%)	16 (48.48%)
	IV	2 (3.64%)	2 (6.67%)	7 (21.21%)

Data are Median (Q1, Q3), Mean ± SD, or N (%).

Abbreviations: NT-proBNP, N-terminal pro-B-type natriuretic peptide; LVEF, left ventricular ejection fraction; LVEDD, left ventricular end-diastolic diameter; LVEDV, left ventricular end-diastolic volume; SV, stroke volume; 6 MWD, 6-min walking distance; MLHFQ, minnesota living with heart failure questionnaire.

### Transcriptomic characteristics of QDBS, YDBS and YDBSFR in IHF

**FIGURE 1:**
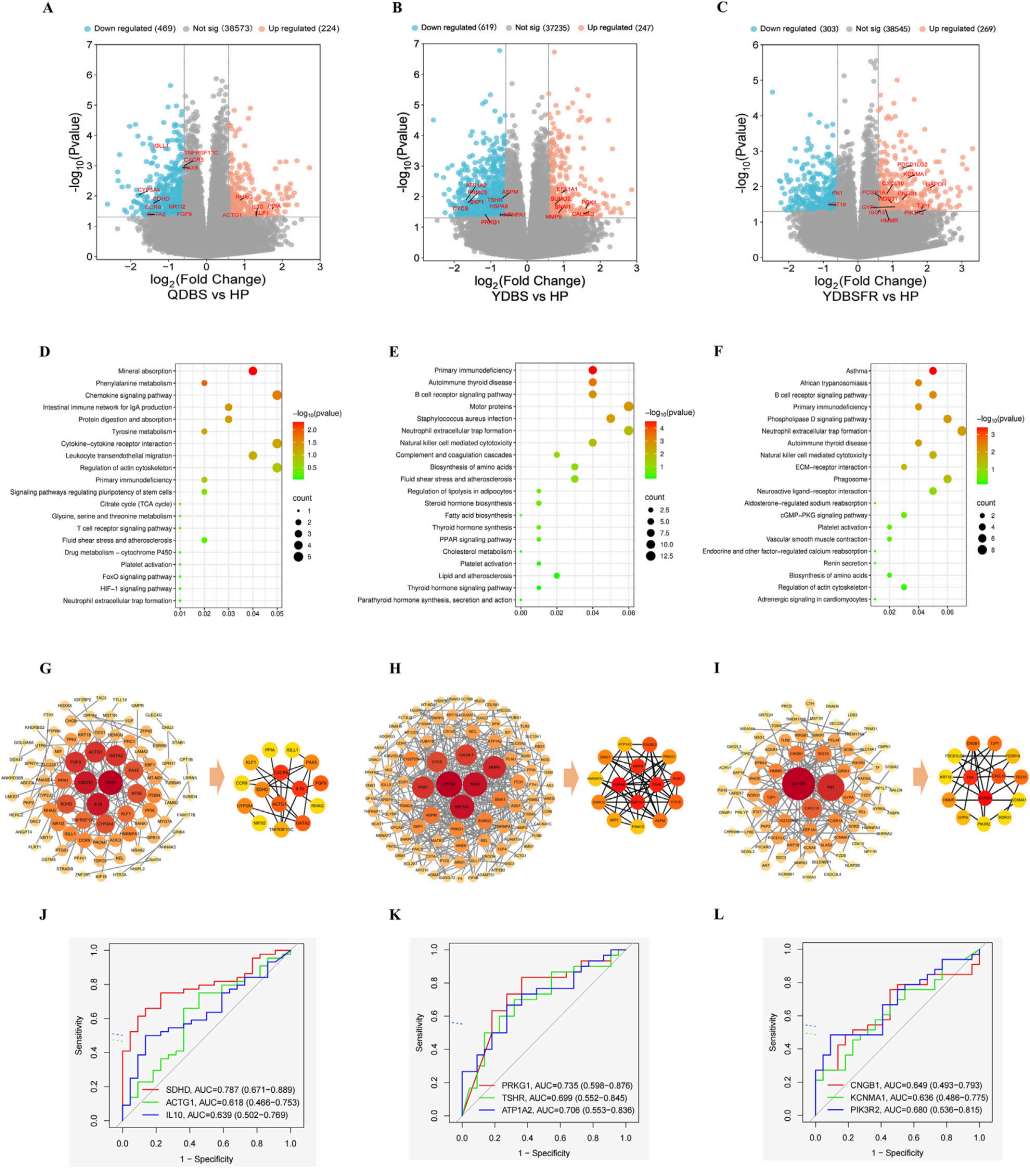
Transcriptomic characteristics of QDBS, YDBS and YDBSFR. **(A–C)** The DEGs volcano maps of HP, QDBS, YDBS and YDBSFR. **(D)** Enrichment pathway of specific DEGs to QDBS. **(E)** Enrichment pathway of specific DEGs to YDBS. **(F)** Enrichment pathway of specific DEGs to YDBSFR. **(G)** PPI analysis of specific DEGs and nodes of algorithm intersection to QDBS. **(H)** PPI analysis of specific DEGs and nodes of algorithm intersection to YDBS. **(I)** PPI analysis of specific DEGs and nodes of algorithm intersection to YDBSFR. **(J)** The ROC curves of SDHD, ACTG1 and IL10 in QDBS. **(K)** The ROC curves of PRKG1, TSHR and ATP1A2 in YDBS. **(L)** The ROC curves of CNGB1, KCNMA1 and PIK3R2 in YDBSFR.

We performed omics tests on the blood samples of 107 IHF patients (44 QDBS, 30 YDBS, 33 YDBSFR) and 22 HP. DESeq2 software was used to analyze transcriptomic differences among QDBS, YDBS, YDBSFR and HP. After excluding the samples with large outliers (3 in the QDBS group, 2 in the YDBS group, and 3 in the YDBSFR group), the results of PCA showed significant differences between HP and the three syndromes (Supplementary Figures S1A–C). Compared with the HP group, QDBS had 693 DEG, of which 224 were significantly upregulated and 469 downregulated, (Figure 1A); YDBS had 866 DEG, of which 247 were significantly upregulated and 619 downregulated (Figure 1B); YDBSFR had 572 DEGs, of which 269 were significantly upregulated and 303 downregulated (Figure 1C). The Venn diagram was used to visualize the common and specific DEGs of each syndrome type. The results showed that there were 73 common DEGs, and the specific DEGs of QDBS, YDBS and YDBSFR were 398, 596 and 342 respectively (Supplementary Figure S1D). The specific DEGs were subjected to KEGG enrichment analysis. The results showed that QDBS was mainly enriched in pathways including leukocyte transendothelial migration, TCA cycle, platelet activation, HIF-1 signaling pathway, and neutrophil extracellular trap formation (Figure 1D). YDBS was mainly enriched in regulation of lipolysis in adipocytes, PPAR signaling pathway, cholesterol metabolism, platelet activation, and thyroid hormone signaling pathway (Figure 1E). YDBSFR was mainly enriched in neutrophil extracellular trap formation, aldosterone-regulated sodium reabsorption, cGMP-PKG signaling pathway, platelet activation, and vascular smooth muscle contraction (Figure 1F).

The PPI network of QDBS-specific DEGs contained 88 nodes and 119 edges; YDBS comprised 165 nodes and 420 edges; YDBSFR consisted of 79 nodes and 114 edges. Nodes represent genes, edges represent associations between genes, and the size and color of the nodes indicate the magnitude of the degree value. In the intersection of MCC, MNC and Degree algorithms, QDBS included 15 DEGs such as CXCR5, PAX5, IL10, SDHD, KLF1, etc. YDBS contained 15 DEGs including EEF1A1, PRKG1, PGK1, TSHR, CYCS, etc. YDBSFR comprised 14 DEGs like FN1, CXCL10, GAPDH, TJP1, CNGB1, etc. Figures 1G–I show the top 100 nodes with QDBS, YDBS and YDBSFR degree values and the core DEGs, respectively.The results of the ROC curve showed the top three AUC values in QDBS were SDHD, IL10 and ACTG1 (Figure 1J); PRKG1, ATP1A2 and TSHR in YDBS (Figure 1K); PIK3R2, CNGB1 and KCNMA1 in YDBSFR (Figure 1L). Compared with HP group, QDBS showed decreased SDHD expression but increased IL10 and ACTG1, YDBS exhibited lower expression of PRKG1, ATP1A2 and TSHR, YDBSFR demonstrated elevated expression of PIK3R2, CNGB1 and KCNMA1. These hub genes possess certain diagnostic value and reliability, possibly serving as key target genes for the three syndrome types.

### Proteomic characteristics of QDBS, YDBS and YDBSFR in IHF

**FIGURE 2:**
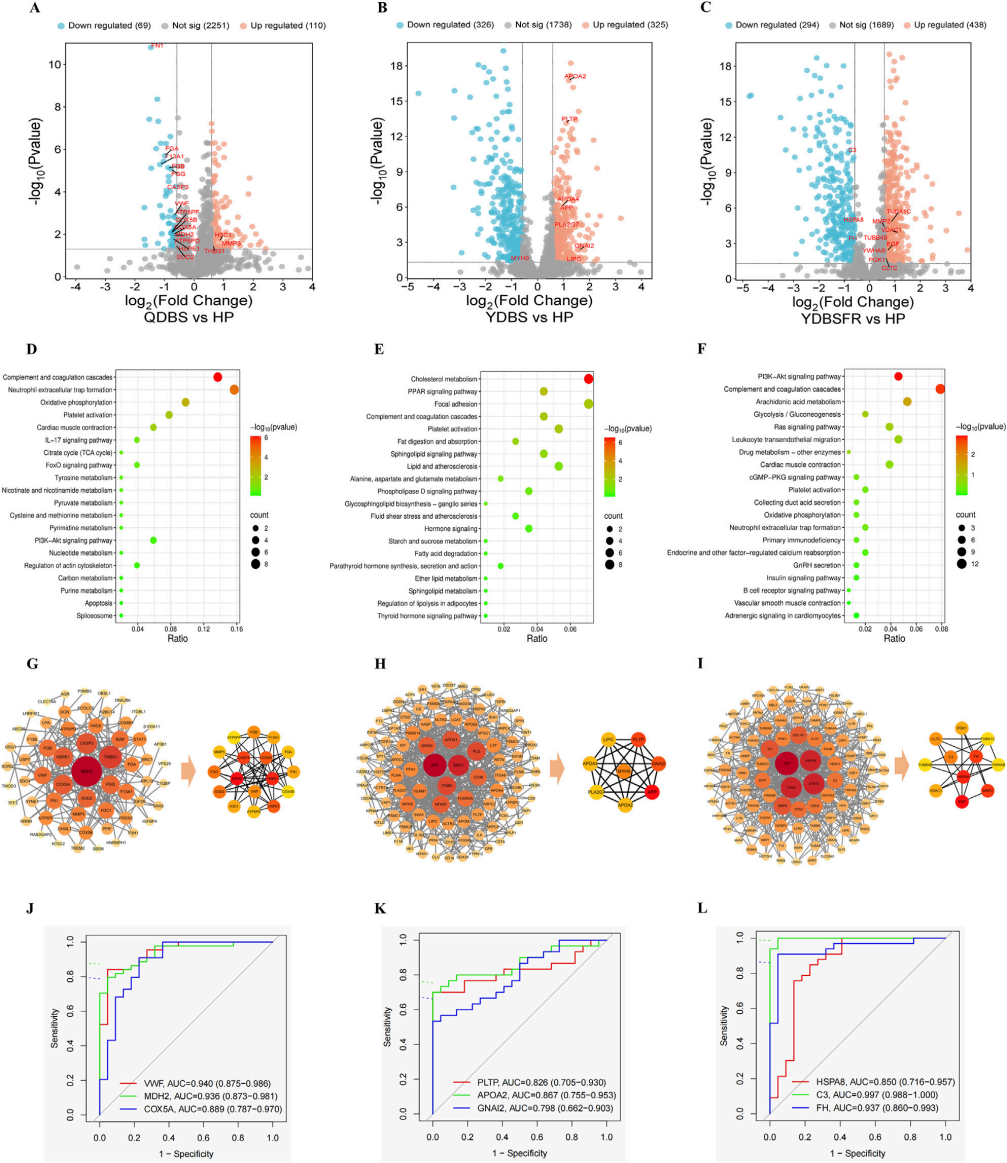
Proteomic characteristics of QDBS, YDBS and YDBSFR. **(A–C)** The DEPs volcano maps of HP, QDBS, YDBS and YDBSFR. **(D)** Enrichment pathway of specific DEPs to QDBS. **(E)** Enrichment pathway of specific DEPs to YDBS. **(F)** Enrichment pathway of specific DEPs to YDBSFR. **(G)** PPI analysis of specific DEPs and nodes of algorithm intersection to QDBS. **(H)** PPI analysis of specific DEPs and nodes of algorithm intersection to YDBS. **(I)** PPI analysis of specific DEPs and nodes of algorithm intersection to YDBSFR. **(J)** The ROC curves of VWF, MDH2 and COX5A in QDBS. **(K)** The ROC curves of PLTP, APOA2 and GNAI2 in YDBS. **(L)** The ROC curves of HSPA8, C3 and FH in YDBSFR.

The proteomic differences of the three syndrome types were analyzed using the DIA method. PCA showed significant differences between HP and the three syndromes (Supplementary Figures S2A–C). Compared with HP group, QDBS had 179 DEPs, with 110 upregulated and 69 downregulated (Figure 2A); YDBS had 651 DEPs, with 325 upregulated and 326 downregulated (Figure 2B); YDBSFR had 732 DEPs, with 438 upregulated and 294 downregulated (Figure 2C). The results of the Venn diagram showed that there were 51 common DEPs, and 85, 192 and 262 specific DEPs in QDBS, YDBS and YDBSFR respectively (Supplementary Figure S2D). The specific DEPs were subjected to KEGG enrichment analysis. The results showed that QDBS was mainly enriched in complement and coagulation cascades, neutrophil extracellular trap formation, Oxidative phosphorylation, platelet activation and TCA cycle (Figure 2D). YDBS was predominantly enriched in cholesterol metabolism, PPAR signaling pathway, platelet activation, regulation of lipolysis in adipocytes and thyroid hormone signaling pathway (Figure 2E). YDBSFR was mainly enriched in complement and coagulation cascades, cGMP-PKG signaling pathway, oxidative phosphorylation, neutrophil extracellular trap formation, and vascular smooth muscle contraction (Figure 2F).

PPI network analysis showed that QDBS-specific DEPs contained 60 nodes and 162 edges, YDBS had 153 nodes and 557 edges and YDBSFR comprised 211 nodes with 721 edges. In the intersection of MCC, MNC and Degree algorithms, QDBS identified 17 hub proteins such as MDH2, THBS1, COX5A, CASP3, VWF, etc. YDBS contained 8 hub proteins including PLTP, APP, GNAI2, APOA2, PLA2G7, etc. YDBSFR comprised 11 hub proteins like HSPA8, MMP2, TUBA1C, C3, EGF, etc. Figures 2G–I show the top 100 nodes with QDBS, YDBS and YDBSFR degree values and the core proteins, respectively. ROC curve analysis showed the top three DEPs with highest AUC values in QDBS were VWF, MDH2 and COX5A (Figure 2J); APOA2, PLTP and GNAI2 in YDBS (Figure 2K); C3, FH and HSPA8 in YDBSFR (Figure 2L). Compared with HP group, QDBS exhibited decreased expression of VWF, MDH2 and COX5A, YDBS showed increased levels of APOA2, PLTP and GNAI2, YDBSFR had lower expression of C3, FH and HSPA8. These hub proteins demonstrated certain diagnostic potential and reliability, and may be the key target proteins for the three syndrome types.

### Metabolomic characteristics of QDBS, YDBS and YDBSFR in IHF

**FIGURE 3:**
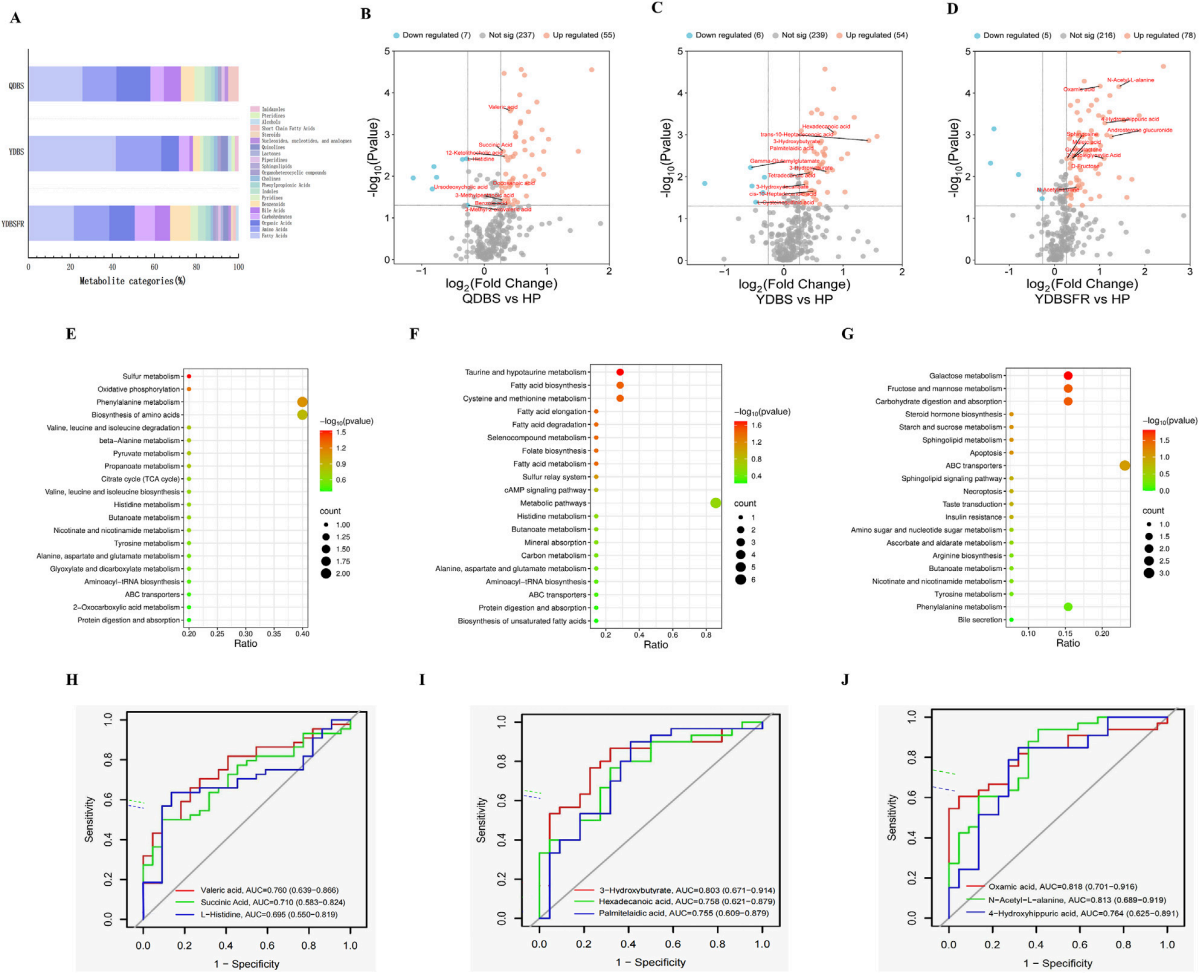
Metabolomic characteristics of QDBS, YDBS and YDBSFR. **(A)** Classification Bar Chart of DMs for QDBS, YDBS and YDBSFR. **(B–D)** The DMs volcano maps of HP, QDBS, YDBS and YDBSFR. **(E)** Enrichment pathway of specific DMs to QDBS. **(F)** Enrichment pathway of specific DMs to YDBS. **(G)** Enrichment pathway of specific DMs to YDBSFR. **(H)** The ROC curves of Valeric acid, Succinic Acid and L-Histidine in QDBS. **(I)** The ROC curves of 3-Hydroxybutyrate, Hexadecanoic acid and Palmitelaidic acid in YDBS. **(J)** The ROC curves of Oxamic acid, N-Acetyl-L-alanine and 4-Hydroxyhippuric acid in YDBSFR.

Targeted metabolite identification and quantitative analysis were performed on blood samples of QDBS, YDBS and YDBSFR using the multi-reaction monitoring mode (MRM), and 205 DMs were identified in plasma. PCA results showed that there were significant differences between HP and the three syndromes (Supplementary Figure S3A–C). Metabolite classification showed that there were 14, 15 and 19 categories in QDBS, YDBS and YDBSFR respectively, predominantly fatty acids, amino acids and organic acids (Figure 3A). Compared with the HP group, QDBS had 62 DMs, of which 55 were significantly upregulated and 7 downregulated (Figure 3B); YDBS showed 60 DMs, of which 54 were significantly upregulated and 6 downregulated (Figure 3C); YDBSFR exhibited 83 DMs, of which 78 were significantly upregulated and 5 downregulated (Figure 3D). The results of the Venn diagram showed that there were 35 common DMs, and 9, 14 and 25 DMs specific to QDBS, YDBS and YDBSFR, respectively (Supplementary Figure S3D). The specific DMs were subjected to KEGG enrichment analysis. The results showed that QDBS metabolic pathways were mainly involved in oxidative phosphorylation, phenylalanine metabolism, biosynthesis of amino acids, pyruvate metabolism, and TCA cycle (Figure 3E). YDBS metabolic pathways were primarily enriched in fatty acid biosynthesis, fatty acid degradation, fatty acid metabolism, and biosynthesis of unsaturated fatty acids (Figure 3F). YDBSFR pathways were mainly associated with carbohydrate digestion and absorption, steroid hormone biosynthesis, apoptosis, and insulin resistance (Figure 3G). The results of the ROC analysis showed the top three DMs with highest AUC values in QDBS were Valeric acid, Succinic Acid and L-Histidine (Figure 3H); 3-Hydroxybutyrate, Hexadecanoic acid and Palmitelaidic acid in YDBS (Figure 3I); Oxamic acid, N-Acetyl-L-alanine and 4-Hydroxyhippuric acid in YDBSFR (Figure 3J). These hub metabolites demonstrated certain diagnostic potential and reliability, possibly serving as key metabolites for the three syndrome types. Specific biomarkers lists, AUC values and KEGG pathways can be found in the Supplementary Material S3.

### Joint analysis of multi-omics

**FIGURE 4:**
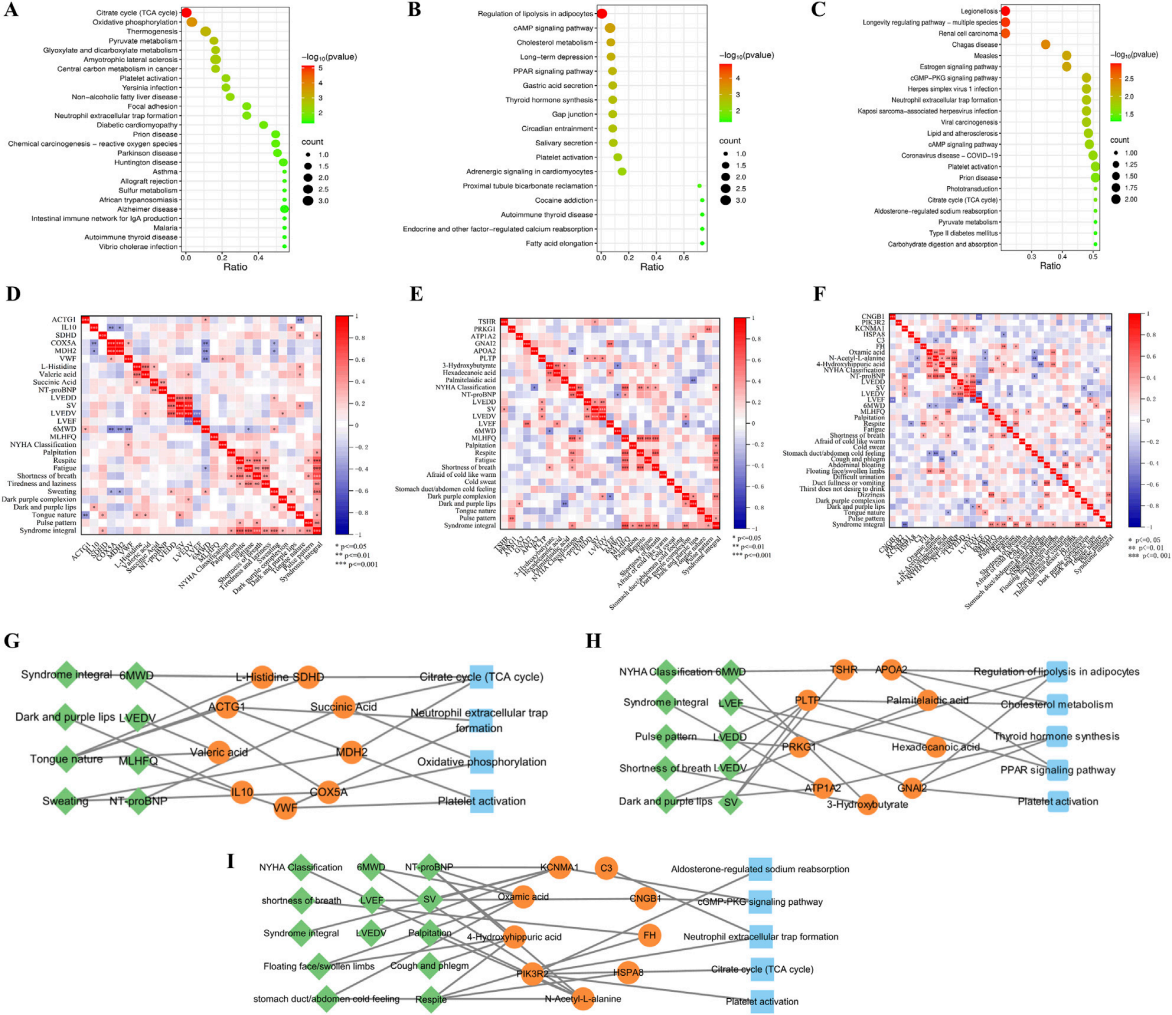
Joint network construction of clinical phenotypes and multi-omics. **(A)** Joint pathway analysis of DEGs, DEPs, and DMs in QDBS. **(B)** Joint pathway analysis of DEGs, DEPs, and DMs in YDBS. **(C)** Joint pathway analysis of DEGs, DEPs, and DMs in YDBSFR. **(D)** Correlation analysis of biomarkers and clinical phenotypes in QDBS. **(E)** Correlation analysis of biomarkers and clinical phenotypes in YDBS. **(F)** Correlation analysis of biomarkers and clinical phenotypes in YDBSFR. **(G)** “Disease-Syndromes-Clinical phenotypes-Biomarkers-Pathways” network of QDBS. **(H)** “Disease-Syndromes-Clinical phenotypes-Biomarkers-Pathways” network of YDBS. **(I)** “Disease-Syndromes-Clinical phenotypes-Biomarkers-Pathways” network of YDBSFR.

Joint pathway analysis revealed 26 significant pathways in QDBS (p < 0.05), mainly enriched in TCA cycle, oxidative phosphorylation, platelet activation and neutrophil extracellular trap formation (Figure 4A), related to energy metabolism, coagulation system and immune-inflammatory response; 17 pathways in YDBS (p < 0.05), primarily enriched in regulation of lipolysis in adipocytes, cholesterol metabolism, PPAR signaling pathway and thyroid hormone synthesis (Figure 4B), associated with lipid metabolism and hormone regulation; and 22 pathways in YDBSFR (p < 0.05) mainly enriched in cGMP-PKG signaling pathway, aldosterone-regulated sodium reabsorption, neutrophil extracellular trap formation, platelet activation, and TCA cycle (Figure 4C), involving signal transduction, hormone regulation, immune-inflammatory response and energy metabolism. Meanwhile, we found that platelet activation was a common enrichment pathway for QDBS, YDBS, and YDBSFR.

### Correlation analysis of key DEGs, DEPs and DMs with clinical phenotypes

To determine the interrelationships between key biomarkers and clinical phenotypes including syndromes and cardiac function-related indicators, Spearman correlation analysis was performed between DEGs, DEPs, DMs and NT-proBNP, LVEF, LVEDD, LVEDV, SV, 6WMD, MLHFQ, symptoms/signs and syndrome scores of the three syndrome types, respectively.The results showed that in QDBS, SDHD and COX5A were significantly correlated with 6MWD, VWF with MLHFQ, Succinic Acid with NT-proBNP, Valeric acid with LVEDV, and multiple biomarkers showing significant correlations with QDBS syndrome (Figure 4D). In YDBS, TSHR and PLTP were correlated with SV, GNAI2 and Hexadecanoic acid with LVEF, APOA2 with NYHA Classification, 3-Hydroxybutyrate with 6MWD, PLTP with LVEDD and LVEDV, and multiple biomarkers significantly associated with YDBS syndrome (Figure 4E). In YDBSFR, CNGB1 was correlated with LVEF, FH and Oxamic acid with 6MWD, KCNMA1 with NT-proBNP, SV and LVEDV, N-Acetyl-L-alanine with NYHA Classification, NT-proBNP and 6MWD, and multiple biomarkers significantly correlated with YDBSFR syndrome (Figure 4F). Correlation analysis results can be found in the Supplementary Material S3.

### Construction of the “Disease-Syndromes-Clinical phenotypes-Biomarkers-Pathways” network

Based on the connections between symptoms, signs, clinical indicators and DEGs, DEPs, DMs and their enriched pathways in QDBS, YDBS and YDBSFR patients, we used Cytoscape visualization software to construct a “Disease-Syndromes-Clinical phenotypes-Biomarkers-Pathways” network, comprehensively revealing the differences of the three syndromes from multiple levels and perspectives. The key biomarkers of QDBS were SDHD, IL10, ACTG1, VWF, MDH2, COX5A, Valeric acid, Succinic Acid and L-Histidine, mainly enriched in TCA cycle, oxidative phosphorylation, platelet activation and neutrophil extracellular trap formation pathways, and significantly correlated with clinical phenotypes including dark and purple lips, tongue nature, 6MWD, NT-proBNP,etc. (Figure 4G). The key biomarkers of YDBS were PRKG1, ATP1A2, TSHR, APOA2, PLTP, GNAI2, 3-Hydroxybutyrate, Hexadecanoic acid and Palmitelaidic acid, mainly enriched in regulation of lipolysis in adipocytes, cholesterol metabolism, PPAR signaling pathway, thyroid hormone synthesis and platelet activation pathways, and significantly correlated with shortness of breath, pulse pattern, LVEF, SV and other clinical phenotypes. (Figure 4H). The key biomarkers of YDBSFR were PIK3R2, CNGB1, KCNMA1, C3, FH, HSPA8, Oxamic acid, N-Acetyl-L-alanine and 4-Hydroxyhippuric acid, mainly enriched in aldosterone-regulated sodium reabsorption, cGMP-PKG signaling pathway, neutrophil extracellular trap formation, TCA cycle and platelet activation pathways, and significantly correlated with clinical phenotypes including stomach duct/abdomen cold feeling, floating face/swollen limbs, NYHA Classification, NT-proBNP, etc. (Figure 4I). The three networks visualize the biological basis of QDBS, YDBS and YDBSFR from multiple levels and perspectives.

### Validation

**FIGURE 5:**
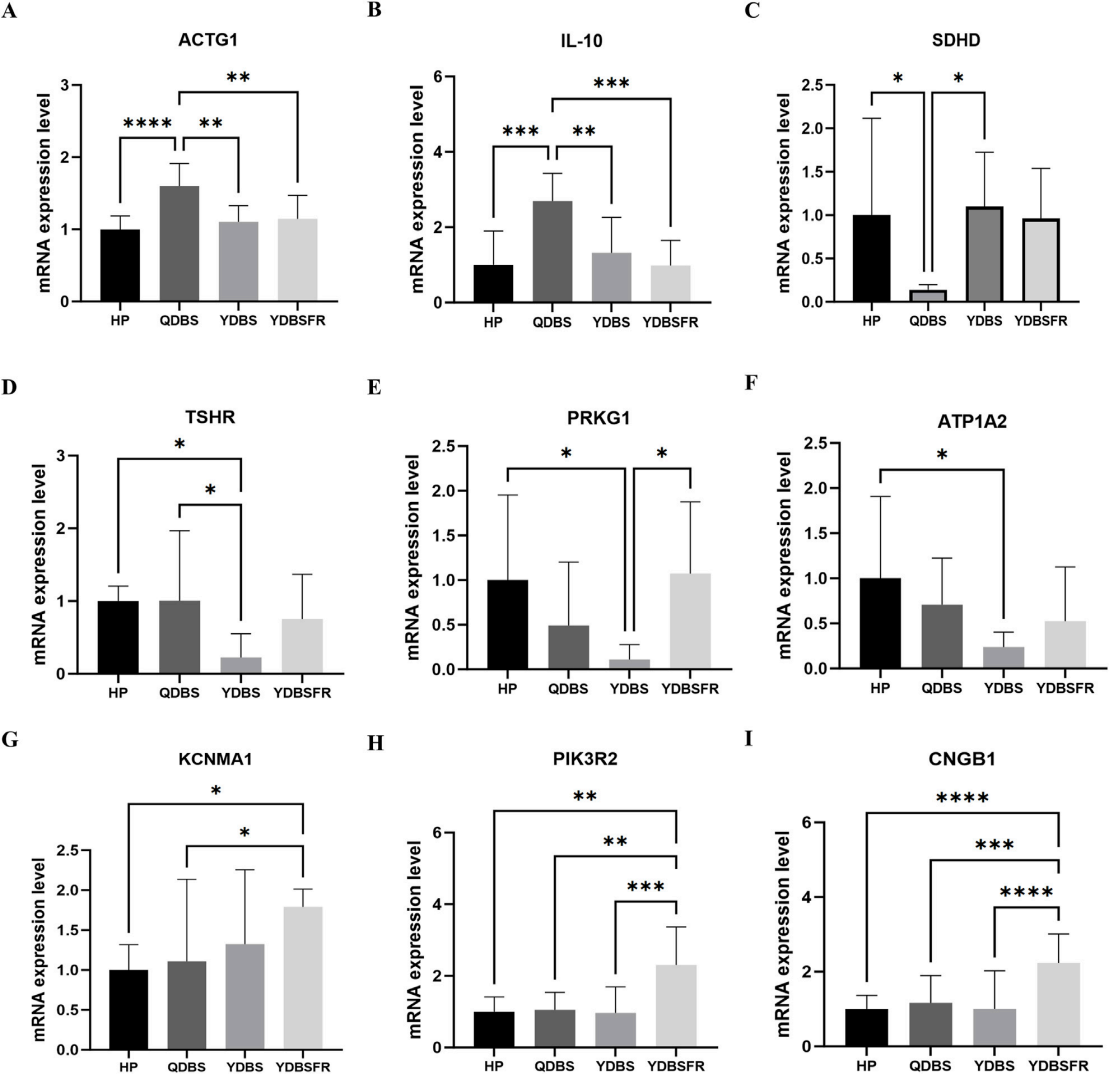
The RT-qPCR validation of candidate biomarkers for QDBS, YDBS and YDBSFR. **(A–C)** The mRNA expressions of ACTG1, IL10 and SDHD in QDBS. **(D–F)** The mRNA expressions of TSHR, PRKG1 and ATP1A2 in YDBS. **(G–I)** The mRNA expressions of KCNMA1, PIK3R2 and CNGB1 in YDBSFR.

We enrolled 30 IHF patients in the validation stage, including 10 QDBS, 10 YDBS and 10 YDBSFR patients, along with 10 HP from the health examination center. The four groups were comparable in age, gender, BMI, heart rate, SBP and DBP (P > 0.05), and the demographic information is shown in Supplementary Table S6. Based on the key gene results from this study, we further validated the DEGs of the three syndromes using RT-qPCR. Compared with HP group, QDBS showed higher expression of ACTG1 and IL10 but lower expression of SDHD (Figures 5A–C), YDBS exhibited decreased expression of TSHR, PRKG1 and ATP1A2 (Figures 5D–F), and YDBSFR demonstrated increased expression of KCNMA1, PIK3R2 and CNGB1 (Figures 5G–I). The expression trends of DEGs in the three syndromes were consistent with the sequencing stage, and all the 9 indicators showed statistical significance compared with HP group (P < 0.05). In addition, ACTG1 and IL-10 in the QDBS group were statistically significant compared with the YDBS and YDBSFR groups, and PIK3R2 and CNGB1 in the YDBSFR group were statistically significant compared with the QDBS and YDBS groups.

**FIGURE 6:**
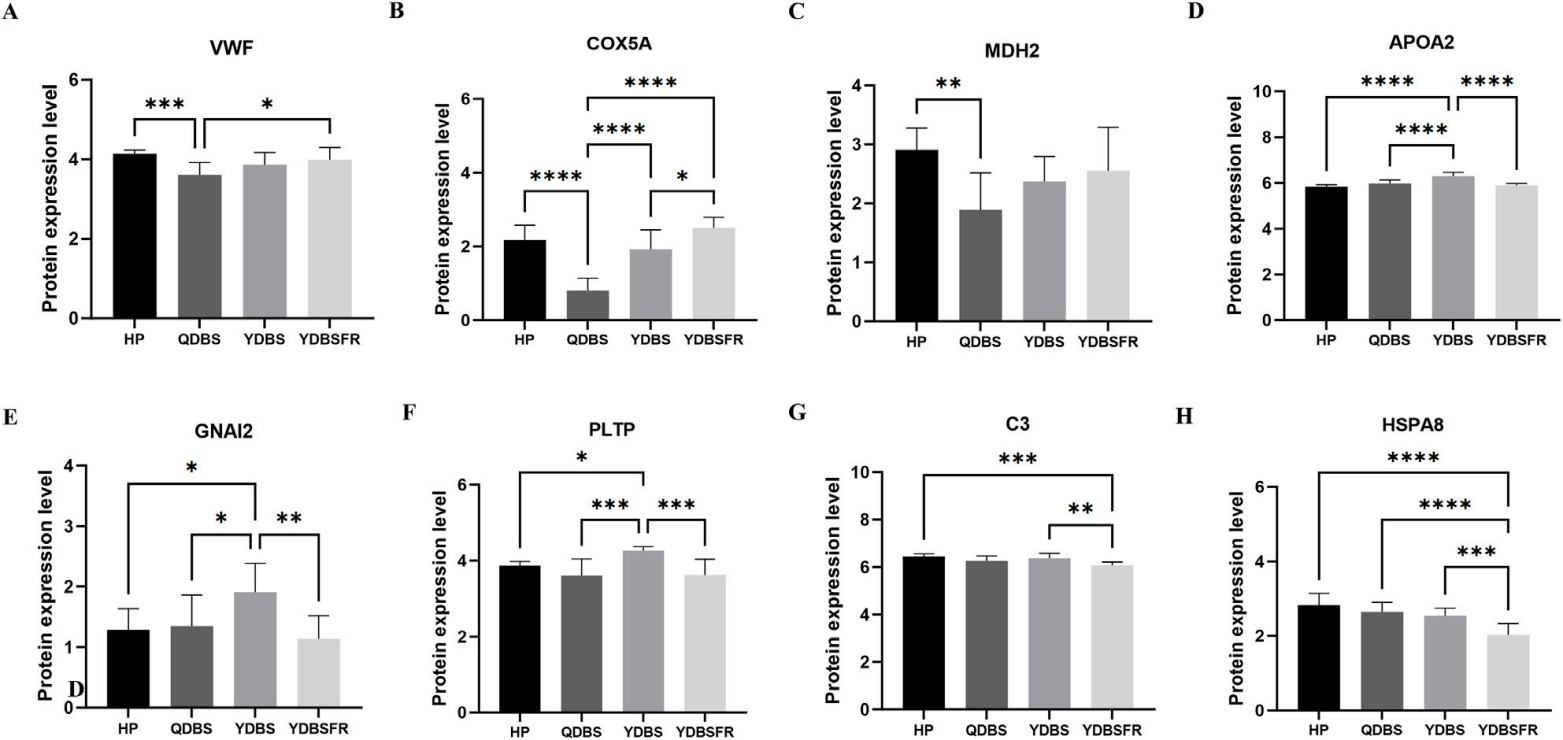
The iPRM validation of candidate biomarkers for QDBS, YDBS and YDBSFR. **(A–C)** The differential expression of VWF, COX5A and MDH2 in QDBS. **(D–F)** The differential expression of APOA2, GNAI2 and PLTP in YDBS. **(G–H)** The differential expression of C3 and HSPA8 in YDBSFR.

Based on the key protein results, we performed validation of DEPs using iPRM targeted proteomics. Compared with HP group, QDBS showed decreased expression of VWF, COX5A and MDH2 (Figures 6A–C), YDBS exhibited higher expression of APOA2, GNAI2 and PLTP (Figures 6D–F), and YDBSFR demonstrated lower expression of C3 and HSPA8 (Figures 6G,H). The expression trends of key DEPs in the three syndromes were consistent with the sequencing stage, and all these indicators were statistically significant (P < 0.05). However, FH was not validated in the samples, possibly due to the physicochemical properties of the selected peptides affecting the mass spectrometry ionization efficiency or the homology of the selected characteristic peptides, resulting in interference or suppression of the mass spectrometry signal.

## Discussion

In this study, we investigated patients with QDBS, YDBS and YDBSFR of IHF, and analyzed patients’ blood samples for clinical indicators, transcriptomics, DIA proteomics, and targeted metabolomics to elucidate the biological basis of the three syndrome types.

### The severity of IHF progressively worsens along the evolution of QDBS, YDBS and YDBSFR

In terms of clinical indicators, we compared the differences in cardiac function-related indicators among the three syndrome types. Along the disease progression from QDBS through YDBS to YDBSFR, NT-proBNP, LVEDD, LVEDV and MLHFQ scores showed significant increasing trends, and LVEF gradually decreased and 6WMD progressively shortened. NYHA classification also differed among syndromes, with QDBS being predominantly class II, YDBS having the highest proportion of class III, YDBSFR also being mainly class III, and YDBSFR showing a higher percentage of class IV patients. In the context of modern medicine, an increasing number of studies have revealed the pathological characteristics of different HF stages through correlations between TCM syndrome types and objective indicators, demonstrating that syndromes are able to reflect the severity of disease to some extent. For example, Sun et al. analyzed clinical characteristic differences in NYHA classification, NT-proBNP levels, MLHFQ, and echocardiography among different HF syndrome types, showing that QDBS represents a relatively stable disease stage, while YSBS syndrome belongs to a more severe stage of the disease process (Sun et al., 2024). Huang et al. demonstrated in a prospective research that as syndromes progress from QDBS to YDBS, there is an increasing trend in NYHA classification and NT-proBNP levels, and both TCM syndrome types and NYHA classification are major factors affecting quality of life in HF patients (Huang et al., 2023). These studies have made important contributions to promoting the objectification of TCM syndrome differentiation in HF. Our results also indicate that QDBS syndrome represents an early stage of HF, and along the pathological progression from QDBS through YDBS to YDBSFR, IHF severity shows a gradually worsening trend.

### Energy metabolism, coagulation system, immune-inflammatory response and QDBS

In this study, through high centrality ranking combined with ROC curve analysis, SDHD, IL10, ACTG1, VWF, MDH2, COX5A, Valeric acid, Succinic Acid and L-Histidine were identified as potential biomarkers of QDBS. Joint pathway analysis revealed that the molecular pathways related to QDBS were TCA cycle, oxidative phosphorylation, platelet activation and neutrophil extracellular trap formation, mainly associated with energy metabolism, coagulation system and immune-inflammatory response. IHD is the main factor in the occurrence of HF. Altered energy metabolism is a typical characteristic of HF, and the resulting energy deficiency causing cardiac overload leads to varying degrees of HF (Schenkl et al., 2023). In TCM theory, Qi has the function of promoting and stimulating, and it is an essential subtle substance to maintain the life activities of human body. Qi promotes the circulation of essence, blood and body fluids, stimulates material metabolism and energy transformation, which shows similarities with mitochondrial oxidative energy supply (Li et al., 2023).

Under normal conditions, to maintain mechanical and electrical activities ensuring normal pumping function, the heart breaks down various substrates and generates large amounts of ATP through mitochondrial TCA cycle and oxidative phosphorylation (OXPHOS) (Li et al., 2024; Lopaschuk et al., 2021). The TCA cycle constitutes the hub of cellular metabolism, serving as the core metabolic pathway for ATP production, and also being the primary cause of mitochondrial dysfunction in failing myocardium. Succinate dehydrogenase (SDH) is the initiating enzyme representing the succinate oxidation respiratory chain, uniquely located in the inner mitochondrial membrane in the TCA cycle. It participates in complex II of the mitochondrial electron transport chain, oxidizing succinate to fumarate which is then converted to malate by fumarate hydratase, and further converted to oxaloacetate by malate dehydrogenase (MDH), combining with acetyl-CoA to form the TCA cycle. In the TCA cycle, the electron transport chain transfers electrons from complexes I and II through OXPHOS to ultimately produce ATP (Martínez-Reyes and Chandel, 2022; Ye et al., 2022). Under the pathological environment of HF, abnormal mitochondrial structure and function inhibit the TCA cycle and OXPHOS processes, leading to reduced ATP production (Brown et al., 2017). Dysfunctional mitochondria cause cell permeability transition, increased reactive oxygen species (ROS) generation, mitochondrial membrane potential collapse, which further induce platelet activation (Choo et al., 2012). Additionally, excessive ROS trigger oxidative stress, exacerbate endothelial cell damage (Scioli et al., 2020), and cause procoagulant factors like von Willebrand factor (vWF) and activated platelets to adhere together to damaged vascular endothelial cells, forming thrombi with the assistance of fibrinogen and others (Tomaiuolo et al., 2017). It has been demonstrated that activated platelets can secrete various cytokines and signaling molecules, directly or indirectly participating in immune regulation processes (Koupenova et al., 2018), promoting the formation of neutrophil extracellular traps (NETs) (Clark et al., 2007). NETs can bind to endothelial cells through vWF and P-selectin, providing a scaffold for platelet, neutrophil and erythrocyte binding, which further leading to fibrin deposition and thrombosis, and aggravating myocardial fibrosis and vascular injury (Dumont et al., 2024; Sørensen and Borregaard, 2016). Our results suggest that QDBS patients may have energy metabolism disorders, with abnormal platelet activation and immune-inflammatory responses occurring on this basis, which interact to form a closed-loop pathological network that further drives the progression of HF.

### Thyroid hormone, lipid metabolism and YDBS

According to the results of multi-omics analysis, the potential biomarkers of YDBS were TSHR, PRKG1, ATP1A2, GNAI2, APOA2, PLTP, 3-Hydroxybutyrate, Hexadecanoic acid and Palmitelaidic acid, and the joint pathways were mainly enriched in thyroid hormone synthesis, regulation of lipolysis in adipocytes, cholesterol metabolism and PPAR signaling pathway, related to hormone regulation, and lipid metabolism. The thyroid gland is the largest endocrine gland in the human body and has a thermogenic effect. Research have shown that symptoms like cold intolerance and cold limbs in Yang deficiency patients may be related to impaired thermogenesis caused by significant downregulation of thyroid hormone receptor expression (Wang and Yao, 2008). Thyroid hormones play an important role in left ventricular remodeling after ischemia, maintaining cardiovascular function and mitochondrial integrity. Hypothyroidism can reduce myocardial contractility and cardiac output, and participate in myocardial fibrosis (Bano et al., 2020; Jabbar et al., 2017). Therefore, it is closely related to the occurrence, progression and poor prognosis of HF. 15%–30% of HF patients have reduced serum triiodothyronine (T3) levels (Lisco et al., 2020), and decreased T3 levels combined with increased TSH levels increase the risk of mortality from HF in cardiovascular disease patients (Neves et al., 2019; Thayakaran et al., 2019).

Under normal conditions, myocardial energy supply is primarily based on fatty acid metabolism, and energy and lipid homeostasis are crucial for normal cardiac structure and function. The maintenance of this homeostasis depends on the coordinated regulation of lipid uptake, oxidation, triglyceride synthesis, lipolysis and lipoprotein secretion (Kliewer et al., 2001). Studies show that HF caused by pressure overload or IHD is often accompanied by imbalance of fatty acid uptake and utilization, leading to abnormal lipid metabolism. This makes the heart more susceptible to lipotoxic cardiac dysfunction (Abdalla et al., 2011; Palomer et al., 2016). Peroxisome proliferator-activated receptor α (PPARα) is located downstream of thyroid hormone receptor and can promote lipid oxidation (Liu et al., 2007). It is also a key molecule downstream of AMPK that regulates myocardial mitochondrial fatty acid oxidation (FAO), playing a crucial role in modulating cardiomyocytes lipid metabolism, including FA uptake and oxidation, TG synthesis, mitochondrial respiratory uncoupling, and transcriptional expression of key enzymes in glucose metabolism (Kaimoto et al., 2017; Yang and Li, 2007). Dysfunction of PPARα can cause excessive FA uptake, FAO inhibition or decreased lipid secretion, leading to lipid accumulation in cardiomyocytes, resulting in contractile dysfunction and consequently leading to the occurrence of HF (Zhou et al., 2000). Our results suggest that YDBS patients may have impaired thyroid hormone synthesis, and reprogramming of myocardial lipid metabolism mediated through PPAR signaling pathway, leading to cardiac systolic-diastolic decompensation that exacerbates the progression of HF.

### Aldosterone hormone, cGMP/PKG signaling pathway and YDBSFR

The potential biomarkers of YDBSFR were CNGB1, KCNMA1, PIK3R2, HSPA8, C3, FH, Oxamic acid, N-Acetyl-L-alanine and 4-Hydroxyhippuric acid, and the combined pathways were mainly enriched in aldosterone-regulated sodium reabsorption and cGMP-PKG signaling pathway, which are related to hormone regulation, signal transduction. YDBSFR is commonly seen in advanced HF. Prolonged heart Yang deficiency damages spleen and kidney Yang qi, causing dysfunction, impaired Qi transformation, dysregulated water metabolism and flooding of the skin, with edema as the main clinical manifestation. Edema is the main clinical feature of extracellular fluid volume expansion, characterized by pathological sodium balance and persistent accumulation of excess water in the intercellular matrix. Aldosterone, secreted from zona glomerulosa cells of the adrenal cortex, is one of the important hormones regulating electrolytes and extracellular fluid volume. It can maintain fluid volume and water balance by promoting sodium reabsorption in distal convoluted tubules and collecting ducts (Weir et al., 2015). When HF occurs, decreased cardiac output, reduced blood pressure and renal perfusion, lead to the activation of RAAS. The rapid increase in aldosterone secretion leading to sodium and water retention, increases blood volume and cardiac preload (Abassi et al., 2022), and also progressive exacerbation of the condition of HF patients by inducing perivascular and interstitial fibrosis leading to ventricular remodeling (Sun and Chen, 2019). Numerous studies have shown that adding aldosterone receptor antagonists such as spironolactone and finerenone on the basis of standard treatment can reduce morbidity and mortality in HF patients (Epstein, 2021a; Epstein, 2021b).

Atrial natriuretic peptide (ANP) can inhibit renin release from juxtaglomerular cells, thereby suppressing the reabsorption of sodium from RAAS and exerting natriuretic, diuretic and vasodilatory effects (Ichiki and Burnett, 2017). ANP binds to guanylate cyclase-A (GC-A) receptor and catalyzes the formation of cyclic guanosine monophosphate (cGMP), which is an important intracellular second messenger whose main signaling mediator is PKG. The cGMP/PKG signaling pathway widely exists in cardiomyocytes and exerts various cardiovascular protective effects including inhibiting cardiomyocyte apoptosis (Numata and Takimoto, 2022). Dysfunction of this pathway is one of the major causes of cardiac remodeling, which can lead to the development of HF. During HF, atrial/ventricular wall dilation and intracardiac volume overload enhance the secretion of natriuretic peptide, leading to increased ANP levels in plasma. However, due to hemodynamic changes or increased degradation and clearance rates of natriuretic peptide, the effects of ANP are attenuated, resulting in reduced ability to inhibit RAAS, while also causing dysregulation of the cGMP/PKG signaling pathway, which contribute to the development of HF (Abassi et al., 2022). In recent years, positive results have been achieved in the treatment of HF with drugs that activate the cGMP-PKG pathway. Sacubitril/valsartan can improve HF by inhibiting neprilysin (NEP) and angiotensin receptors, enhancing the protective effects of the cGMP/PKG pathway on cardiomyocytes. Additionally, the cGMP/PKG signaling pathway can also be activated through the nitric oxide (NO)-soluble guanylate cyclase (sGC) pathway. Vericiguat, as an sGC stimulator, upregulates cGMP expression, thereby restoring blunted cGMP/PKG signaling in HF (Nakai et al., 2016). Our results suggest that YDBSFR patients may be associated with RAAS overactivation, increased aldosterone secretion causing sodium and water retention, and dysregulation of the cGMP/PKG signaling pathway leading to cardiomyocyte apoptosis and cardiac remodeling. In addition, we found that the key biomarkers of YDBSFR were also enriched in neutrophil extracellular trap formation, platelet activation, and TCA cycle signaling pathways. This indicates that in advanced HF, multiple interacting mechanisms including circulatory dysfunction, volume overload, ventricular remodeling and energy depletion collectively lead to disease progression, existing in a vicious pathological cycle of “yang deficiency failing to transform qi and promote water, with blood stasis and water retention mutually aggravating each other”.

### Platelet activation mediates the whole process of IHF

IHD is prone to the formation of arterial thrombi through platelet activation and aggregation, which is a major cause of the development of HF (Kim et al., 2016). Large-scale cohort studies have confirmed that compared with healthy individuals, patients with HF have significant platelet activation, and the activation levels correlates with the severity of cardiac function to a certain extent (Gibbs et al., 2001). Activated platelets can exacerbate HF progression by mediating inflammatory responses through lipid mediator release and neutrophil interactions, promoting myocardial fibrosis. After the rupture of atherosclerotic plaques, the exposure of the vascular subcutaneous matrix leads to the release of various prothrombotic factors, which promote platelet adhesion and activation. Lipids derived from platelet activation can trigger pro-inflammatory responses through intercellular interactions, leading to vascular inflammation (Peng et al., 2018). Meanwhile, platelets participate in neutrophil recruitment and activation by releasing soluble mediators and adhesion molecules. Their interaction causes neutrophil activation and retention at thrombus sites, activating complement and coagulation cascades, resulting in thrombotic inflammation, which is not only a key driver of atherosclerotic plaque progression but also promotes inflammatory factor infiltration in cardiomyocytes and extracellular matrix deposition, leading to myocardial fibrosis and ventricular remodeling (Stark and Massberg, 2021). In addition, hemodynamic changes during IHF progression increase catecholamines in the circulating and activate RAAS, which continuously activate platelets through positive feedback (Larsson et al., 2000), forming a closed-loop pathological network of “platelet activation-inflammation-fibrosis-cardiac dysfunction”. Our results demonstrate that platelet activation exists throughout the pathological evolution of different IHF syndrome types, and may participate in the pathological process of HF by mediating inflammatory responses and myocardial fibrosis.

### Molecular pathophysiological basis of TCM syndromes

According to TCM, Qi has the function of promoting and warming. Qi can promote the normal operation of blood in the blood vessels. Qi deficiency results in symptoms such as shortness of breath and fatigue, which correspond to impaired mitochondrial energy metabolism, which reduces ATP production. Qi deficiency impairs blood propulsion, leading to QDBS, which aligns with our findings that the abnormal mitochondrial function in patients with QDBS leads to platelet activation and directly or indirectly participates in the immune modulation of the organism to further lead to thrombus formation. Yang qi can warm the body, and Yang deficiency will result in fear of cold and cold limbs, which is consistent with impaired heat production due to impaired thyroid hormone synthesis and abnormal lipid metabolism. Chronic Yang deficiency impairs Qi transformation, leading to edema due to dysregulated fluid metabolism, which aligns with aldosterone-mediated sodium retention. YDBSFR predominantly manifests in advanced HF, characterized by the complex interplay of Yang deficiency, blood stasis, and fluid retention, indicating severe disease progression. This is highly correlated with our observed pathological mechanisms in advanced HF, including circulatory dysfunction, volume overload, ventricular remodeling, and bioenergetic exhaustion. Our study deeply explored the correspondence between TCM theories and molecular pathophysiology, and realized the connection between classical TCM disease mechanisms and modern pathophysiology.

In summary, this study takes modern biomedicine as a new perspective, integrates the concept of syndrome in TCM with the pathophysiological mechanisms of modern medical, correlates the biological basis of TCM syndromes with clinical phenotypes, deeply explores the biological essence of TCM syndromes in IHF. We have constructed the “Disease-Syndromes-Clinical phenotypes-Biomarkers-Pathways” network, which comprehensively reveals the differences of the three syndromes from multiple perspectives. The biomarkers identified in this study demonstrate multi-dimensional academic values in the future clinical translation of IHF in diagnosis, treatment, and drug development. In the field of diagnosis, a molecular biomarker system for syndrome classification based on multi-omics can be constructed, and multi-indicator joint diagnostic kit can be developed to combine the subjective identification of Chinese medicine with biomarkers, so as to promote the quantitative fusion of Chinese and Western medicine diagnostics, and improve the scientific objectivity of syndrome classification. In terms of treatment and drug development, the energy metabolism of QDBS patients can be regulated through the development of mitochondria-targeted drugs combined with TCM to tonify qi and invigorate blood. The lipid metabolism and thyroid function of YDBS patients can be improved by the use of PPARα agonists or thyroid receptor agonists combined with TCM that warm yang and invigorate blood. The use of aldosterone receptor antagonists can be optimized in patients with YDBSFR by the development of cGMP-PKG pathway agonists and combining with Chinese herbs that warm yang and promote fluid to improve volume overload. Based on the biomarkers discovered in this study, reverse screening of the active ingredients of Chinese medicine compound can realize the precision of integrated Chinese and Western medicine treatment, which provides a new theoretical basis and practical path for the targeted treatment and drug development of IHF, which has important clinical translational significance and academic values. In addition, in this study, an intuitive and visualized “Disease-Syndrome-Clinical phenotypes-Biomarker-Pathway” network was constructed through multi-dimensional information integration to reveal the systematic biological basis of the TCM syndromes of ischemic heart failure (IHF). The network of different syndrome types combines the holistic perspective of syndrome differentiation in TCM with the molecular mechanism of Western precision medicine, demonstrating the causal relationship between molecular markers and TCM syndromes. It provides objective support at the molecular level for TCM theory and is conducive to promoting the transformation of syndrome diagnosis from qualitative description to precise quantification.

There are some limitations in the implementation process of this study. Firstly, since the participants in this study were collected in Henan region during the same period, there was an imbalance in the sample sizes of the three syndrome types, which was considered to be possibly related to the distribution characteristics of syndromes. The QDBS syndrome type is more commonly seen in the early and relatively stable stage of IHF, with a slow progression of the disease and a larger patient base, while YDBS and YDBSFR are in the middle and late stages of IHF, with more severe conditions resulting in limited cases meeting the inclusion and exclusion criteria. Secondly, all the participants in this study were from Henan region, and the biomarker profiles of the syndrome patterns were regionally specific. In the future, we will conduct animal models and cellular experiments to further validate the identified potential biomarkers and signaling pathways to ensure the biological reliability of the results. Multicenter and large-sample studies will be carried out, including people from different regions and ethnic groups, to verify the universality of biomarkers and construct a spectrum of TCM syndromes - biomarkers covering the characteristics of a broader population. In addition, the study did not strictly control the drug use of the patients and there were confounding factors such as comorbidities. Different types of drugs and comorbidities may affect the accuracy of the association between biomarkers and syndromes to a certain extent. In the future, a standardized drug use record and monitoring process will be established. Methods such as propensity score matching will be adopted to balance the interference of different drugs on biomarkers. Meanwhile, subgroups of comorbidities will be set up to analyze the specific impact of comorbidities on the biomarker network of different syndrome types, in order to eliminate confounding factors and clarify the core characteristics of syndrome-related biomarkers.

## Conclusion

This study explores the biological basis of QDBS, YDBS and YDBSFR in IHF and their associations with clinical phenotypes from a modern biomedical perspective. The three syndrome types show significant correlations with multiple clinical phenotypes, and the IHF condition progressively aggravated with the evolution of QDBS-YDBS-YDBSFR. QDBS may be associated with energy metabolism disorders, immune-inflammatory responses and overactivation of the coagulation system. YDBS may have impaired thyroid hormone synthesis and reprogramming of myocardial lipid metabolism through the PPAR signaling pathway. YDBSFR is in the stage of deterioration of advanced HF with the interaction of multiple mechanisms including volume overload, ventricular remodeling and energy depletion. Platelet activation persists throughout IHF progression. This study investigates the potential mechanisms of TCM syndromes and the correlation between biomarkers and clinical phenotypes from the perspective of modern biomedicine, providing references for the objectification research of TCM syndromes.

## Data Availability

The original contributions presented in the study are publicly available. This data can be found here: https://www.ncbi.nlm.nih.gov/geo/query/acc.cgi?acc=GSE303117.

## Abbreviations

QDBS, Qi Deficiency and Blood Stasis Syndrome; YDBS, Yang Deficiency and Blood Stasis Syndrome; YDBSFR, Yang Deficiency and Blood Stasis with Fluid Retention Syndrome; HP, Healthy Persons; IHF, ischemic heart failure; TCM, Traditional Chinese medicine; DEGs, differentially expressed genes; DEPs, differentially expressed proteins; DMs, differentially metabolites; LVEDD, left ventricular end-diastolic diameter; LVEDV, left ventricular end-diastolic volume; LVEF, left ventricular ejection fraction; SV, stroke volume; MLHFQ, Minnesota living with heart failure questionnaire; NT-proBNP, N-terminal pro-B-type natriuretic peptide; NYHA, New York Heart Association; VIP, Variable importance projection; PCA, Principal component analysis; PLS-DA, Partial least squares discriminant analysis; PPI, Protein-Protein Interaction Network; ROC, Receiver Operating Characteristic; AUC, Area under the receiver operating curve; RT-qPCR, Real-time Quantitative Polymerase Chain Reaction; iPRM, Intelligent Parallel Reaction Monitoring; 6MWD, 6-min walking distance.
