# Supplementary material for: Exploring the biological basis for the identification of different syndromes in ischemic heart failure based on joint multi-omics analysis
Source: Front Pharmacol. 2025 Jul 28;16:1641422. doi: 10.3389/fphar.2025.1641422 (PMC12337011; doi:10.3389/fphar.2025.1641422)
Supplement: Supplementary file 1 [file Supplementaryfile1.docx]

**Supplementary Materials**

**Table S1. Diagnostic criteria for syndrome types**

| Guidelines for Diagnosis and Treatment of Chronic Heart Failure in Chinese Medicine (2022) | |
| --- | --- |
| Qi deficiency and blood stasis Syndrome | Main symptoms: shortness of breath/ respite, fatigue, palpitation.  Secondary symptoms: (1) Tiredness and laziness, easy to exert oneself in activities; (2) Sweating; (3) low voice; (4) The complexion/lips are dark purple.  Tongue and pulse: Purple-dark tongue (or with ecchymosis, petechiae or sublingual varices), normal-sized tongue with white coating, and deep, thin or weak pulse. |
| Yang deficiency and blood stasis Syndrome | Main symptoms: shortness of breath/ respite, fatigue, palpitation.  Secondary symptoms: (1) Afraid of cold or like warm; (2) Stomach duct/abdomen/ waist/limbs feeling cold; (3) The body feels cold, accompanied by sweating; (4) The complexion/lips are dark purple.  Tongue and pulse: Purple-dark tongue (or with ecchymosis, petechiae or sublingual varices), fat tongue or teeth marks, thin, deep and slow/weak pulse. |
| Yang deficiency blood stasis with fluid retention | Main symptoms: shortness of breath/ respite, fatigue, palpitation.  Secondary symptoms: (1) Afraid of cold or like warm; (2) Sweating; (3) Stomach duct/abdomen/waist/limbs feeling cold; (4) Cough and phlegm; (5) Abdominal bloating; (6) Floating face or swollen limbs; (7) Difficult urination; (8) Duct fullness or vomiting; (9) Thirst does not desire to drink; (10) Dizziness; (11) The complexion/lips are dark purple.  Tongue and pulse: Lubricated or greasy tongue, smooth pulse. |
| The diagnosis can only be recognized if the above syndromes have 2 main symptoms and 2 secondary symptoms respectively, combined with the tongue and pulse. | |

**Table S2. Transcriptomic research**

| Blood RNA extraction and detection | Venous blood samples (2.5 ml) were collected from fasting subjects in the morning and placed in PAXgeneTM tubes (PreAnalytiX, China). After gentle inversion 8-10 times for thorough mixing and proper labeling, the tubes were stored at 4°C for 24 hours according to the manufacturer's instructions, then transferred to -80°C freezers. Samples were shipped on dry ice to Novogene Co., Ltd (Beijing) for mRNA extraction and analysis.  Total RNA was extracted using PAXgene Blood miRNA Kit. RNA concentration was measured by Agilent 5400 bioanalyzer. Agarose gel electrophoresis was performed to check RNA purity and potential DNA contamination. RNA integrity was accurately determined using Agilent 5400 bioanalyzer. |
| --- | --- |
| Library construction, quality control and sequencing | 1. Library construction: Using total RNA as starting material, polyA-tailed mRNA was enriched by Oligo(dT) magnetic beads. The enriched mRNA was randomly fragmented by divalent cations in Fragmentation Buffer. In the M-MuLV reverse transcriptase system, random oligonucleotides were used as primers to synthesize the first-strand cDNA with fragmented mRNA as template, and the RNA strand was degraded using RNaseH. Subsequently, the second cDNA strand was synthesized using dNTPs as raw materials in DNA polymerase I system. About 370~420 bp of purified double-stranded cDNA was screened by using AMPure XP beads after end repair, A-tail addition and sequencing junction ligation steps, and further amplified by PCR, and then the PCR product was purified again by using AMPure XP beads, and the library was finally obtained. 2. Quality control: Preliminary quantification was performed using Qubit 2.0 Fluorometer. After dilution, insert size was detected by Agilent 2100 bioanalyzer, and the effective library concentration (>2 nM) was accurately quantified by qRT-PCR to ensure the library quality. 3. Sequencing: After passing quality control, libraries were pooled according to the effective concentration and target downstream data volume, then sequenced on Illumina NovaSeq 6000 to generate 150 bp paired-end reads to obtain the sequence information of the fragments to be tested. |
| Bioinformatics analysis | 1. Data quality control: Image data of sequencing fragments were obtained from high-throughput sequencer and converted to sequence data (reads) by CASAVA base recognition. After filtering, reads with undetermined base information, reads with splice, and reads with Qphred ≤ 20 bases accounting for ≥ 50% of the whole read length were removed. Q20, Q30 and GC contents of cleaned data were calculated to obtain high-quality dataset.   (2)Sequence alignment to the reference genome: Reference genome and gene annotation files were downloaded from genome website. HISAT2 v2.0.5 was used to build genome index and align the paired-end clean reads with the reference genome.  (3) Quantification of gene expression levels: Feature Counts (1.5.0-p3) was used to calculate the reads mapped to each gene. FPKM (fragments per kilobase of transcript per million mapped reads) was calculated based on gene length to estimate gene expression levels. |
| Data processing and analysis | Differential gene expression and enrichment analysis: The number of genes in the samples was normalized using DESeq2 software (1.20.0) , and the fold change (FC) was calculated. The P-values were adjusted to control the false discovery rate using the method of Benjamini and Hochberg. KEGG enrichment analysis of the DEGs was performed by clusterProfiler (3.8.1) software to obtain the advanced function and utility of the gene biological system. |

**Table S3. Data-independent-acquisition-based proteomic study**

| Plasma Protein Extraction and Quality Control | Morning fasting venous blood was collected into EDTA anticoagulant tubes, gently inverted 8-10 times for thorough mixing, labeled, and centrifuged at 3000×g for 15 min at 4°C. The supernatant was transferred to labeled Eppendorf tubes, flash-frozen in liquid nitrogen, stored at -80°C, and shipped on dry ice to Novogene Co., Ltd (Beijing) for protein extraction and analysis. High-abundance proteins were depleted using Bio-Rad ProteoMiner beads enrichment kit (Bio-Rad, USA). Protein concentration was measured using Bradford Protein Assay Kit (Beyotime, China). Add the sample to the DB proteolytic solution, add trypsin and TEAB buffer for enzymatic digestion reaction, and wash it through a C18 desalination column. Samples were analyzed by liquid chromatography-tandem mass spectrometry (LC-MS/MS). |
| --- | --- |
| LC-MS/MS detection in DIA mode | Firstly, prepare mobile phase A (100% water, 0.1% formic acid) and B (80% acetonitrile, 0.1% formic acid). The lyophilized powder was dissolved in 10 µL of solution A, centrifuged at 14,000g for 20 min at 4°C, and 200 ng of supernatant was taken for LC-MS analysis. Then a C18 pre-column 174500 (5 mm × 300 μm, 5 μm, Thermo Fisher, USA) and a C18 analytical column ES906 (PepMap TM Neo UHPLC 150 µm x 15 cm, 2 μm, Thermo Fisher, USA) column incubator were heated in an ultra-high-performance liquid chromatography (UHPLC) system at 50 °C. Mass spectrometry analysis was performed using Orbitrap Astral Mass Spectrometer (Thermo Fisher, USA) with ESI ion source, ion spray voltage set at 1.9 kV and ion transfer tube temperature at 290°C. The mass spectrometry analysis used data-dependent acquisition mode, with primary mass spectrometry scanning range of m/z 380-980 and resolution set to 240,000 (at m/z 200). To ensure sufficient signal intensity, AGC was set to 500%, precursor ion window to 2 Th, DIA windows to 300, NCE to 25%, secondary ion m/z acquisition range to 150-2000, subion resolution Astral to 80,000, and maximum injection time to 3 ms. Finally converted to raw mass spectrometry data (.raw format). |
| Protein identification and analysis | Using DIA-NN software with the homo_sapiens_uniprot_2023_10_18_Swissprot.fasta (20,427 sequences) database, we performed in-depth search and analysis of raw files. The search parameters were strictly set within a mass tolerance of 10 ppm for precursor ions and 0.02 Da for fragment ions. In addition, cysteine was modified by alkylation, methionine was oxidatively modified, and N-terminal modifications included acetylation, loss of methionine, and loss of methionine + acetylation. One missed cleavage site was allowed at most. DIA-NN was used to strictly filter results, retaining only peptide-spectrum matches (PSMs) and proteins with >99% confidence. Retention time correction was performed with the iRT added to the sample and the precursor ion Qvalue cutoff value was set to 0.01. False Discovery Rate (FDR) validation was performed to remove peptides and proteins exceeding 1% FDR. |
| Data processing and analysis | T-test was used for statistical analysis of protein quantification results. Proteins with FC >1.5 or FC <0.67 and P <0.05 were defined as differentially expressed proteins. The KEGG Pathway database (www.kegg.jp/kegg/pathway.html) was used to analyze significant enrichment of DEGs in specific pathways. |

**Table S4. Detection procedures for targeted metabolomics**

| Blood sample extraction | Morning fasting venous blood was collected in EDTA anticoagulant tubes, thoroughly mixed, and centrifuged. The supernatant was centrifuged at 3000×g for 10 min at 4°C,then collected into Eppendorf cryopreservation tubes, labeled, and stored at -80°C. 300 μL of 80% aqueous methanol was added to 100 μL of plasma samples, mixed well, and centrifuged at 4°C at 12,000 rpm for 15 minutes after 15 minutes on ice. 50 μL supernatant was mixed with 150 μL derivatization reagent (50 μL 160mM 3-NPH, 50 μL 120mM EDC, 50 μL 8% pyridine), and derivatized at 40°C for 40 min. 90 μL supernatant was mixed with 10 μL internal standard solution and analyzed by LC-MS. In order to evaluate the stability of the mass spectrometry platform during the whole experiment, an equal amount of all plasma samples was mixed into QC samples for analysis. |
| --- | --- |
| Standard Curve Establishment | Accurately weigh each metabolite standard to prepare mixed linear master batch, then dilute with methanol to obtain working solutions at different concentrations. Prepare the isotope internal standard (IS) solution of specified concentration, and mix well to obtain the internal standard solution. The linear, IS and QC master batches and working solutions were stored in a refrigerator at -20℃. LC-MS analysis was performed for standard concentration series, using standard-to-IS concentration ratio as x-axis and peak area ratio as y-axis to evaluate linearity of the standard solutions . |
| LC-MS/MS detection and analysis | The metabolites were detected and quantified using UHPLC-MS/MS system (ExionLC™ AD UHPLC-QTRAP 6500+, AB SCIEX Corp., Boston, MA, USA) . The separation was performed using Waters HSS T3 column (2.1×150mm) with a controlled injection volume of 2 uL, a column temperature of 40 °C, a flow rate of 0.3 mL/min, a mobile A phase of 0.1% formic acid in water; and 50% acetonitrile-isopropanol as the mobile B phase. The chromatographic gradient was set as follows: 95% A, 1 min; 60-95% A, 7 min; 5-60% A, 25 min; 5-95% A, 27.1 min; 95% A,30 min. The mass spectrometry was scanned using an electrospray ionization source with positive/negative ion multiple reaction monitoring (MRM), and the temperature of the ion source was set at 550°C, the voltage of the ion source at 4500V/-4500V, the sheath gas at 35 psi, auxiliary gas at 50 psi, and collision gas at 55 psi. |
| Qualitative and quantitative analysis of metabolites | After ionization, samples enter the triple quadrupole mass spectrometer system. In this system, the Q1 screens the precursor ions with specific mass-to-charge ratios (m/z). Subsequently, the precursor ions were put into the Q2 fragmentation cell to become subion with different mass-to-charge ratios, which were again screened by the Q3 quadrupole to obtain subion with specific mass-to-charge ratios. The metabolites were identified by comprehensive evaluation of multiple parameters, and the MRM mode of the triple quadrupole was applied to achieve accurate quantitative evaluation of the compounds based on the comparison of the peak area of the Q3 (subion) with the standard curve. |
| Data processing and analysis | Metabolomics data were preprocessed and transformed using meta X software, and Variable Importance in the Projection (VIP) of metabolite variables was obtained by principal component analysis (PCA) and partial least squares discriminant analysis (PLS-DA). The statistical significance of metabolite differences between the two groups was assessed using T-test, and the fold change (FC) values were calculated. DMs were screened with VIP>1, P<0.05 and FC>1.2 or FC<0.833. Identified metabolites were annotated using KEGG database (https://www.genome.jp/kegg/pathway.html), the HMDB database (https://hmdb.ca/metabolites) and the LIPIDMaps database (http://www.lipidmaps.org/) . |

**Table S5. Primer information**

| Gene |  | Primer | Gene ID | Amplicon Size(bp) |
| --- | --- | --- | --- | --- |
| ACTG1 | Forward | CCGAGCCGTGTTTCCTTCC | 71 | 142 |
|  | Reverse | GCCATGCTCAATGGGGTACT |  |  |
| IL-10 | Forward | GACTTTAAGGGTTACCTGGGTTG | 3586 | 112 |
|  | Reverse | TCACATGCGCCTTGATGTCTG |  |  |
| SDHD | Forward | ATTTCTTCAGGACCGACCTATCC | 6392 | 86 |
|  | Reverse | CAGCCTTGGAGCCAGAATG |  |  |
| TSHR | Forward | GGAATGGGGTGTTCGTCTCC | 7253 | 78 |
|  | Reverse | GCGTTGAATATCCTTGCAGGT |  |  |
| PRKG1 | Forward | CTTGGAGCTGTCGCAGATCC | 5592 | 81 |
|  | Reverse | TCTTTGATGATGCAACTGTCCTT |  |  |
| ATP1A2 | Forward | CACCACCGAAGATCAGTCTGG | 477 | 132 |
|  | Reverse | CGCTTAGACACGGAGATGTTC |  |  |
| KCNMA1 | Forward | CGGACGCTCAAGTACCTGTG | 3778 | 79 |
|  | Reverse | AGCCATTGTTAATCTTCTGGGC |  |  |
| PIK3R2 | Forward | AAAGGCGGGAACAATAAGCTG | 5296 | 85 |
|  | Reverse | CAACGGAGCAGAAGGTGAGTG |  |  |
| CNGB1 | Forward | GGACCCCTCGGAAGACCAA | 1258 | 92 |
|  | Reverse | CTCAGGATTCGGTTCTGGTTC |  |  |

**Table S6. Validation of demographic characteristics of QDBS, YDBS and YDBSFR**

| Index | QDBS（N=10） | YDBS（N=10） | YDBSFR(N=10) | HP(N=10) |
| --- | --- | --- | --- | --- |
| Age, years | 71.1±4.41 | 66.7±7.33 | 70.6±7.41 | 68.7±2.54 |
| Male, N (%) | 6（60.00%） | 7（70.00%） | 5（50.00%） | 6（60.00%） |
| BMI,kg/cm^2^ | 25.66±4.26 | 23.36±2.04 | 24.08±3.39 | 24.17±2.11 |
| HR,beat per minute | 68.9±8.72 | 67.7±8.38 | 65.1±8.44 | 64.6±8.14 |
| Seated SBP, mmHg | 130.3±12.27 | 131±13.86 | 122.3±12.57 | 120.5±11.68 |
| Seated DBP, mmHg | 69.3±10.01 | 73.1±9.65 | 67±6.60 | 73.1±8.90 |

Data are Mean ± SD or N (%) .

Abbreviations: BMI, body mass index;HR, heart rate;SBP, systolic blood pressure; DBP, diastolic blood pressure.
