# Supplementary material for: Exploring the biological basis for the identification of different syndromes in ischemic heart failure based on joint multi-omics analysis
Source: Front Pharmacol. 2025 Jul 28;16:1641422. doi: 10.3389/fphar.2025.1641422 (PMC12337011; doi:10.3389/fphar.2025.1641422)

Fig. S1 Transcriptomic characteristics of QDBS, YDBS and YDBSFR. (A-C) The PCA score classification results of HP, QDBS, YDBS and YDBSFR. (D) DEGs Venn diagram of HP, QDBS, YDBS and YDBSFR.


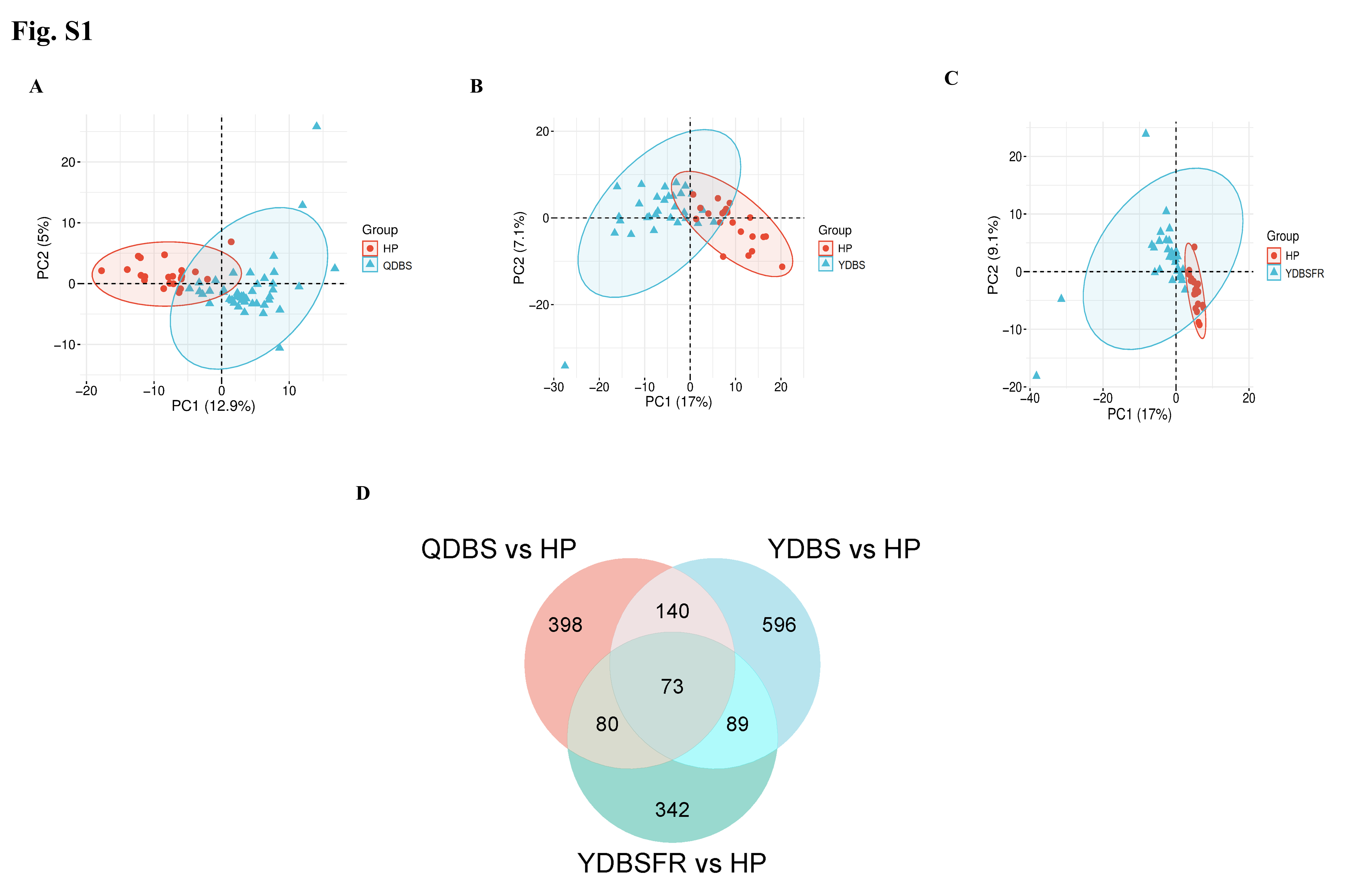


Fig. S2 Proteomic characteristics of QDBS, YDBS and YDBSFR. (A-C) The PCA score classification results of HP, QDBS, YDBS and YDBSFR. (D) The DEPs Venn diagram of HP, QDBS, YDBS and YDBSFR.


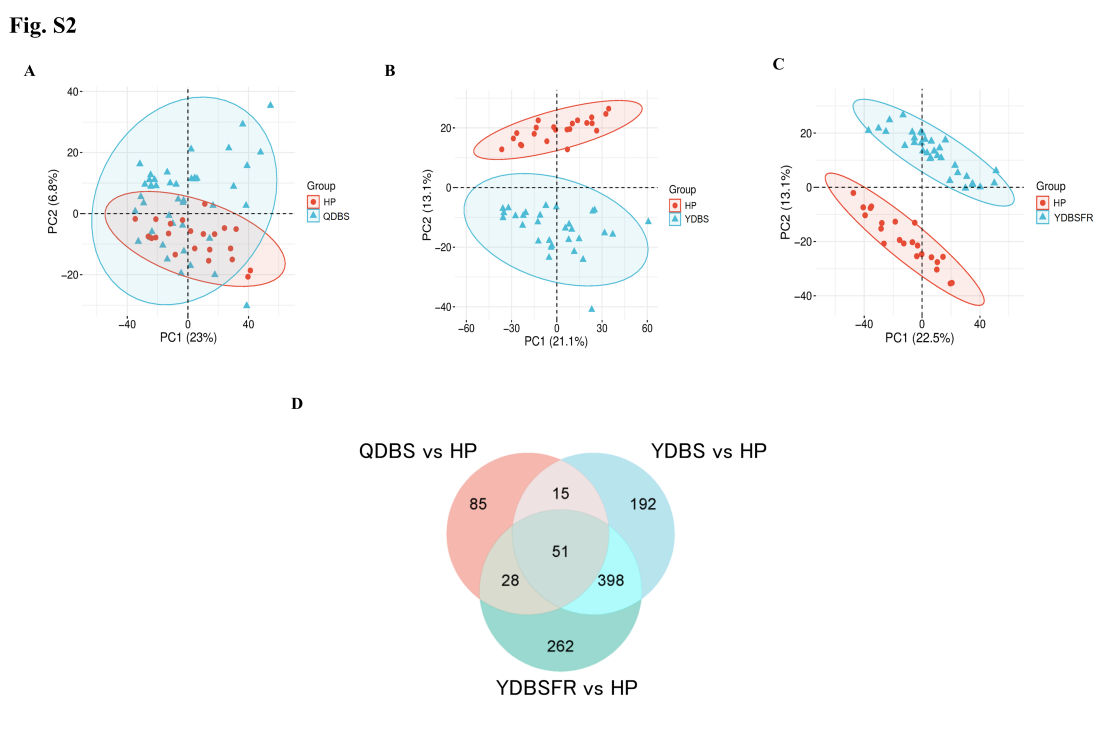


Fig. S3 Metabolomic characteristics of QDBS, YDBS and YDBSFR. (A-C) The PCA score classification results of HP, QDBS, YDBS and YDBSFR. (D) The DMs Venn diagram of HP, QDBS, YDBS and YDBSFR.


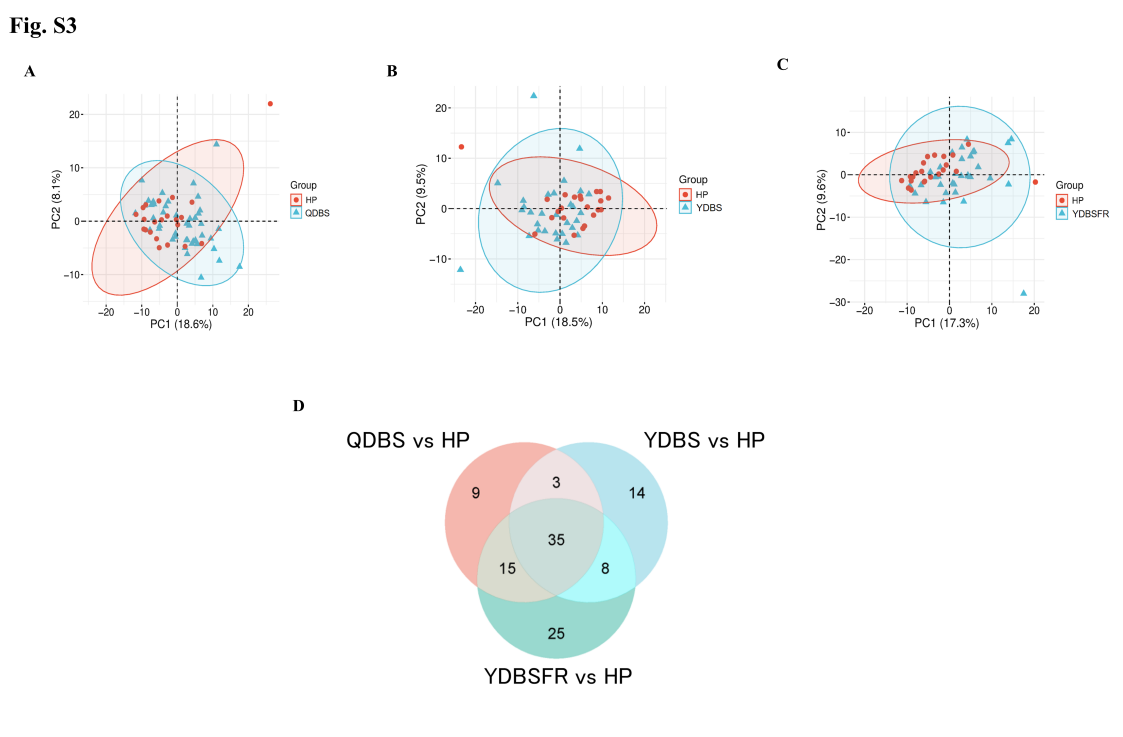

Supplement: Supplementary file 2 [file Supplementaryfile2.docx]
